# Integrated computational investigation of *Glycosmis pentaphylla*-derived compounds against major venom proteins of *Daboia russelii*

**DOI:** 10.3389/fmolb.2026.1906533

**Published:** 2026-09-25

**Authors:** Sana S. Hussain, Danie J. Kingsley

**Affiliations:** School of Bioscience and Technology, Vellore Institute of Technology, VIT University, Vellore, India

**Keywords:** antivenom, extract-derived compounds, *Glycosmis pentaphylla*, molecular docking, molecular dynamics simulation, snake venom

## Abstract

Snakebite envenomation remains a significant neglected tropical disease, particularly in South Asia, where *Daboia russelii* (Russell’s viper) causes substantial morbidity and mortality. Current antivenom therapy is limited by poor efficacy against local tissue damage, high production costs, restricted toxin specificity, and adverse immune reactions, highlighting the need for alternative or adjunct therapeutic strategies. Using an integrated computational and experimental approach, phytocompounds identified from *Glycosmis pentaphylla* leaf extracts were evaluated as potential inhibitors of major venom proteins. A total of 32 compounds identified by GC-MS were screened against phospholipase A_2_ (PLA_2_), snake venom metalloproteinases (SVMPs), snake venom serine proteases (SVSPs), L-amino acid oxidase (LAAO), and snake C-type lectin-like proteins (SNACLECs) using molecular docking. Representative high-affinity protein-ligand complexes were further evaluated by 100 ns molecular dynamics simulations. Pharmacokinetic, toxicity, protein-protein interaction, and pathway enrichment analyses were also performed to assess the therapeutic potential of the identified compounds. Preliminary experimental validation was conducted using trypsin-casein protease inhibition, skim milk agar protease inhibition, and erythrocyte membrane stabilization assays. Compared with N-acetyl cysteine (NAC), β-sitosterol, 3-chloro-5-cholestene, and vitamin E exhibited stronger binding affinities and more stable interactions with multiple venom proteins. Molecular dynamics analyses (RMSD, RMSF, radius of gyration, solvent-accessible surface area, and hydrogen bonding) further supported the stability of the selected protein-ligand complexes. Pharmacokinetic and toxicity predictions indicated acceptable drug-likeness and comparatively safe toxicity profiles for most selected compounds. STRING-based protein-protein interaction and pathway enrichment analyses revealed associations with inflammatory, coagulation, oxidative stress, and extracellular matrix-related pathways involved in venom pathology. The *G. pentaphylla* extract demonstrated concentration-dependent protease inhibition (56.86% at 5 mg/mL), reduced caseinolytic activity (28.19% inhibition), and significant erythrocyte membrane stabilization (39.23%), providing preliminary experimental support for the computational predictions. Overall, these findings identify *G. pentaphylla*-derived phytocompounds as potential lead compounds for future antivenom development and provide a computational foundation for subsequent venom-specific biochemical and *in vivo* validation toward safer and more affordable adjunct therapies for snakebite management.

## Introduction

Snakebite envenomation continues to be a significant public health problem in tropical and subtropical regions, especially in South Asia, where prevalence of morbidity and mortality is high (Alirol et al., 2010; Gopalakrishnan et al., 2022; Gutiérrez et al., 2017; Jayawardana et al., 2020). Of the venomous species, the Russell’s viper (*Daboia russelii*) is an important venomous snake responsible for severe local and systemic effects such as coagulopathy, hemorrhage, tissue necrosis, and renal failure. These effects occur through various venom proteins including phospholipase A_2_ (PLA_2_), snake venom metalloproteinases (SVMPs), serine proteases (SVSPs) (Chudzinski-Tavassi, 2022), L-amino acid oxidases (LAAOs), and C-type lectin-like proteins (Snaclecs) (Arlinghaus and Eble, 2012; Pathan et al., 2017), which also contribute to the complex pathophysiology of envenomation (Chaiyabutr et al., 2020; Latinović et al., 2020; Srinivasan et al., 2024; Tan et al., 2018).

Traditional antivenom therapy is the mainstay of treatment, but is usually limited in its response to neutralize local tissue damage and unwanted immune reaction (Alshammari, 2022; Silva et al., 2022). The search therefore extends to other therapies that could address venom proteins more organically and be based on plant inhibitors, which have molecular targets for action (Adrião et al., 2022; Hussain and Kingsley, 2024; Panda and Ehsan, 2018; Pithayanukul et al., 2009). Traditional medicinal plants are well-known for their ethnopharmacological treatments for snakebite, as they are rich with bioactive extracts derived from their plants with potential inhibitory action (Khandokar et al., 2021).

*Glycosmis pentaphylla* (Retz.) DC., which belongs to the family Rutaceae, is traditionally used in traditional folk medicinal systems for its anti-inflammatory, antimicrobial, and antitoxic activities. Its leaves contain various phytoconstituents with many different antioxidant and enzyme-inhibitory effects, such as alkaloids, flavonoids, terpenoids, and phenolics (Khandokar et al., 2021; Murugan et al., 2020). For *Glycosmis pentaphylla* leaf extracts, they were prepared in methanol, petroleum ether, and hexane solvents (maceration extraction) and the phytochemical constituents were identified using Gas Chromatography-Mass Spectrometry (GC-MS) analysis.

Herein, we utilized PyRx (AutoDock Vina)-based molecular docking for GC-MS profiling of extract-derived compounds (some isolated in samples) onto key venom protein targets of *D. russelii* to assess their binding affinities and interaction with selected peptide-related structures. Nine venom proteins were screened in the first round of phytochemicals targeted for venom in this study, which included PLA_2_ isoforms (PDBID: 1DPY, 1VIP, 2PVT, 2PYC), SVSPs (PDBID: 2S9B, 3SBK), SVMP (PDBID: 2E3X), LAAO (PDBID: 3KVE), and Snaclec (PDBID: 3UBU). Based on their docking affinity and biological relevance, four leading protein-ligand complexes (1VIP, 2PYC, 2S9B, and 2E3X) were selected for 100 ns molecular dynamics (MD) simulation using GROMACS, for further characterization of complex stability and binding behaviour under physiological conditions. Our integrated computational pipeline offers a better understanding of the venom protein-inhibitory potential of *G. pentaphylla* extract-based compounds and strengthens their identification and exploitation as alternative lead/adjunct in snakebite therapy.

While *G. pentaphylla* has previously been studied for its myriad of medicinal activities, to our knowledge, the current study represents the first systematic exploration of the compounds derived from it for antivenom activity. Using three different leaf extracts from bioactive constituents, we investigated their inhibitory potential against a wide range of venom proteins in *D. russelii*. Notably, this is the first work to link molecular docking and molecular dynamics simulations to five high-impact toxin classes (phospholipase A_2_, snake venom serine protease, metalloproteinase, L-amino acid oxidase, and C-type lectin-like proteins) and it represents an overall view regarding the inhibitory potential of *G. pentaphylla* leaf extracts. This multi-targeted, *in silico* strategy enhances emerging understanding of plant-derived antivenom treatments, providing new avenues for safer, cost-effective, and broad-spectrum therapeutic strategies against snake envenomation. More importantly, the knowledge presented in this work will build a strong scientific basis for subsequent *in vitro* and *in vivo* validation studies in order to expedite the translation of the computational predictions into viable therapeutics that have a real social impact.

## Materials and methods

### Ligand choice and preparation

Based on a previous Gas Chromatography-Mass Spectrometry (GC-MS) analysis of *G. pentaphylla* leaf extracts in our previous study, extract-based compounds were chosen for molecular docking. All the major bioactive extract-derived compounds detected were extracted in SDF format from the PubChem database, where each InChIKey was used. Translating the structures to PDBQT (Open Babel v3.1.1), geometry optimization was carried out with MMFF94 force field for steric interaction minimization, while ensuring the favorable conformation for docking (Ayodele et al., 2023; Mun et al., 2022).

### Protein selection and preparation

Based on toxicity, nine venom-targeted proteins related to Russell’s viper envenomation were identified for use in envenomation and coded for their availability in the Protein Data Bank (PDB). The proteins selected were as follows:

- Phospholipase A2 (PLA2): 1DPY, 1VIP, 2PVT, 2PYC
- Snake Venom Serine Protease (SVSP): 2S9B, 3SBK
- Snake Venom Metalloproteinase (SVMP): 2E3X
- L-Amino Acid Oxidase (LAAO): 3KVE
- Snake C-type Lectin-like Protein (SNACLEC): 3UBU

All PDB structures were taken from RCSB Protein Data Bank and checked visually with a PyMOL package (v2.5.2). Water was removed with ligands and ions, and hydrogen atoms added as needed. Active sites were deduced from the presence of co-crystallized ligands or from literature reports.

### Molecular docking using PyRx

Molecular docking was performed using PyRx v0.8, incorporating the AutoDock Vina docking engine. The crystal structures of venom proteins were retrieved from the Protein Data Bank (PDB) and prepared by removing water molecules and non-essential heteroatoms while retaining catalytically important metal ions and cofactors. Ligand structures were energy-minimized and converted to PDBQT format along with the prepared protein structures before being imported into the PyRx virtual screening interface (Dallakyan and Olson, 2015). Docking grids were defined around the experimentally reported active-site residues of each protein, and the exhaustiveness parameter was set to 8 to ensure efficient conformational sampling (Dallakyan and Olson, 2015). For each ligand, the best-ranked binding pose was selected based on the lowest binding affinity and favorable interaction profile. Protein-ligand interactions, including hydrogen bonds, hydrophobic contacts, and interacting amino acid residues, were analyzed and visualized using Discovery Studio Visualizer (Ayodele et al., 2023; Mun et al., 2022).

### Selection of ligands and proteins for MD simulation

Molecular dynamics simulations are computationally intensive; therefore, representative protein-ligand complexes were selected based on predefined criteria. Initially, all 32 phytoconstituents were docked against nine venom-associated proteins. The complexes selected for MD simulations were chosen considering (i) high docking affinity, (ii) representation of the major toxin families involved in *D. russelii* envenomation (PLA_2_, SVSP and SVMP), (iii) high-quality crystal structures suitable for long simulations, and (iv) their established biological roles in venom-induced pathology. Consequently, four representative complexes (1VIP, 2PYC, 3S9B and 2E3X) were subjected to 100 ns MD simulations. A standard benchmark compound NAC, which has anti-PLA2 effect, is taken as positive controls for both docking and simulation studies (Leão-Torres et al., 2021; Sunitha et al., 2011; Wang et al., 2018).

### Molecular dynamics simulation in GROMACS

The protein-ligand complexes were characterized by 100 ns MD simulations based on GROMACS (v2022.2). Ligand topologies were produced by the CHARMM General Force Field (CGenFF) in SwissParam server. The CHARMM36 force field was also involved in the simulation of the protein topology generation (Mun et al., 2022; Ogunyemi et al., 2025). The complex was solvated in a triclinic box by the TIP3P water molecules with 10 Å range in separation. Na^+^ or Cl^−^ ions are added to neutralize the system (Mun et al., 2022; Ogunyemi et al., 2025).

Simulation protocol:

- Energy Minimization: 50,000 steps via steepest descent.
- Equilibration:
  o NVT (constant volume) for 100 ps.
  o NPT (constant pressure) for 100 ps.
- Production Run: 100 ns with periodic boundary conditions.

Temperature (300 K) and pressure (1 bar) were sustained by V-rescale thermostat and Parrinello-Rahman barostat respectively.

Trajectory analyses performed:

- RMSD: Root Mean Square Deviation
- RMSF: Residue flexibility
- Rg: Radius of gyration
- SASA: Solvent accessible surface area
- Hydrogen Bond Analysis

### Experimental validation of computational predictions

#### Trypsin-casein protease inhibition assay

The protease inhibitory activity of *G. pentaphylla* leaf crude extract was evaluated using a trypsin-casein digestion assay as a model system for snake venom serine proteases. Briefly, 50 μL of trypsin solution (0.5 mg/mL in 0.1 M Tris–HCl buffer, pH 8.0) was mixed with 50 μL of plant extract at concentrations ranging from 1 to 5 mg/mL and pre-incubated at 37 °C for 2 min. Subsequently, 100 μL of 0.5% casein solution prepared in the same buffer was added and the reaction mixture was incubated at 37 °C for 10 min. Proteolysis was terminated by adding 50 μL of 10% trichloroacetic acid (TCA), followed by incubation for 5 min and centrifugation at 3,000 rpm for 5 min. An aliquot (100 μL) of the supernatant was transferred to a clean tube and mixed with 100 μL of Bradford reagent. After incubation for 5 min at room temperature, absorbance was measured at 595 nm. Trypsin with casein served as the positive control, while casein without trypsin served as the negative control. Protease inhibition was calculated relative to the positive control.

#### Skim milk agar protease inhibition assay

The protease inhibitory activity of the extract was further assessed using a skim milk agar plate assay. Sterile skim milk agar plates were prepared and wells were aseptically created. Trypsin solution was used as the positive control, distilled water as the negative control, and plant extract alone served as the extract control. For the test group, equal volumes of trypsin solution and plant extract were mixed and pre-incubated prior to loading into the wells. The plates were incubated at 37 °C for 24 h and examined for caseinolytic zones. The diameter of the clear hydrolysis zone surrounding each well was measured in millimetres, and percentage inhibition was calculated relative to the positive control.

#### Erythrocyte membrane stabilization assay

The membrane stabilizing activity of *G. pentaphylla* extract was evaluated using an osmotic stress-induced erythrocyte hemolysis model. Fresh human blood was collected from consenting participants in EDTA-coated tubes was centrifuged, and erythrocytes were washed three times with phosphate-buffered saline (PBS, pH 7.4). A 2% erythrocyte suspension was prepared in PBS. For the assay, 400 μL of distilled water, 50 μL of extract solution (1–5 mg/mL), and 50 μL of erythrocyte suspension were mixed and incubated at 37 °C for 1 h. The mixtures were centrifuged at 1,500 rpm for 5 min, and the absorbance of the supernatant was measured at 540 nm. Distilled water-treated erythrocytes served as the positive control, while erythrocytes suspended in PBS served as the negative control. Membrane stabilization activity was calculated from the reduction in hemoglobin release relative to the positive control.

### Statistical analysis

All experiments were performed in triplicate (n = 3) and except where otherwise indicated (n = 4) and results were expressed as mean ± standard deviation (SD). Statistical analyses were performed using GraphPad Prism software. Differences among groups were evaluated using one-way analysis of variance (ANOVA) followed by Dunnett’s multiple comparison test, with the positive control used as the reference group. Differences were considered statistically significant at P < 0.05. Normality testing, confidence intervals, and effect size analyses were not performed because of the limited sample size.

### Pharmacokinetic, toxicity, protein-protein interaction and pathway enrichment analysis

#### Pharmacokinetic and drug-likeness prediction

The phytocompounds that showed the best performance during molecular docking and molecular dynamics simulation, including β-sitosterol, vitamin E, 3-chloro-5-cholestene, squalene, and 6-nitro-2-methylpyrrolo [2,3-g] quinoline, were further evaluated for their pharmacokinetic behaviour and drug-likeness properties. N-acetyl cysteine (NAC) was included as the reference standard for comparison. Canonical SMILES for each compound were obtained from the PubChem database and submitted to the SwissADME web server for *in silico* prediction of physicochemical and pharmacokinetic parameters. Parameters such as gastrointestinal (GI) absorption, blood-brain barrier (BBB) permeability, lipophilicity, water solubility, bioavailability score, and compliance with Lipinski’s rule of five were analysed. In addition, molecular weight, hydrogen bond donor and acceptor counts, topological polar surface area (TPSA), and LogP values were assessed to determine the overall drug-likeness and therapeutic suitability of the selected compounds (Daina et al., 2017; Lipinski et al., 2001).

#### Toxicity prediction using ProTox-II

The toxicity profiles of the selected phytocompounds and the reference compound NAC were predicted using the ProTox-II web server. Canonical SMILES of the ligands were used as input for estimating oral toxicity (LD50), toxicity class, hepatotoxicity, carcinogenicity, mutagenicity, cytotoxicity, and immunotoxicity. Toxicity categories were interpreted according to the Globally Harmonized System (GHS) classification. Compounds falling within toxicity Classes IV-VI were considered to possess relatively lower toxicity and were therefore regarded as more suitable candidates for further therapeutic investigation (Banerjee et al., 2018; Drwal et al., 2014).

#### Protein-protein interaction and pathway enrichment analysis

To better understand the biological significance of the venom-associated targets, protein-protein interaction (PPI) analysis and pathway enrichment studies were carried out using the STRING database. Genes known to be associated with major pathological events observed during snake envenomation, including inflammation, hemorrhage, coagulation abnormalities, oxidative stress, platelet dysfunction, and extracellular matrix degradation, were selected based on published literature and their established roles in venom pathology. The selected targets included PLA2G2A (Bickler, 2020; Resiere et al., 2022), MMP2, MMP9 (Asega et al., 2020; Kini and Koh, 2016), (F2, F10) (Kini, 2005; Teixeira et al., 2019), (SERPINE1, PLAT) (Frabitore et al., 2022; Yamashita et al., 2014), (TNF, IL6) (Liu et al., 2026; Rao et al., 2024), (AKT1, MAPK1) (Bhattacharya et al., 2013; Bickler, 2020), and PTGS2 (Olivo et al., 2007; Teixeira et al., 2025). *Homo sapiens* was chosen as the reference organism to analyse host-response pathways affected during venom toxicity. STRING analysis was performed using a high-confidence interaction score threshold of 0.700, while disconnected nodes were excluded to obtain a more biologically relevant interaction network (Cavalcante et al., 2023).

Further functional enrichment analysis was performed within the STRING platform to identify significantly enriched Kyoto Encyclopaedia of Genes and Genomes (KEGG) pathways, Reactome pathways, and Gene Ontology (GO) biological processes associated with the selected targets. Particular attention was given to pathways involved in inflammatory signalling, complement and coagulation cascades, platelet activation, oxidative stress, and extracellular matrix remodelling, as these mechanisms are closely linked to the pathological manifestations of Russell’s viper envenomation.

## Results

### Ligand selection and preparation

A total of 32 extract-derived compounds identified through GC-MS analysis of *G. pentaphylla* leaf extracts were selected for *in silico* evaluation (File documents in Supplementary Materials). These included 14 extract-derived compounds from the methanolic extract (MME), 13 from the petroleum ether extract (MPE), and 5 from the hexane extract (MHE). All structures were retrieved from the PubChem database, converted into the PDBQT format, and subjected to geometry optimization using the MMFF94 force field. The finalized set of ligands was stable and suitable for molecular docking analysis.

### Protein selection and preparation

Nine venom-associated proteins related to envenomation of Russell’s viper (*D. russelii*) were prepared for docking experiments. The identified proteins included phospholipase A2 (PLA_2_: 1DPY, 1VIP, 2PVT, 2PYC), snake venom serine proteases (SVSP: 3S9B, 3SBK), snake venom metalloproteinase (SVMP: 2E3X), L-amino acid oxidase (LAAO: 3KVE), and snake C-type lectin-like protein (SNACLEC: 3UBU). All PDB structures were optimized by removal of water molecules, ions, and non-essential heteroatoms, followed by addition of hydrogen atoms. The active sites were defined according to the co-crystallized ligands or literature-reported catalytic residues, ensuring biologically relevant docking interactions.

### Molecular docking analysis

*Glycosmis pentaphylla* methanol (MME, 14 extract-derived compounds), petroleum ether (MPE, 13 extract-derived compounds), and hexane (MHE, 5 extract-derived compounds) extracts were successfully docked against nine venom-associated proteins of *D. russelii* with AutoDock Vina in PyRx for all thirty-two extract-derived compounds. The ligands with highest scores for each protein from each extract are given in the Supplementary Tables 2–4, the 2D diagram of interactions are depicted in Supplementary Figures 2A–I, 3A–I, 4A–I, 5A–I, 6A–I, 7A–I, 8A–I, 9A–I, 10A–I. The binding affinities recorded of the ligands ranged from −3.9 to −9.3 kcal/mol, for which some of the extract-derived compounds showed even greater affinity as compared to the standard reference N-acetylcysteine (NAC), which ranged from modest affinities (−4.0 to −5.0 kcal/mol) are also depicted in Table 1; Figures 1A–I.

**TABLE 1:** Docking scores (kcal/mol) and interacting residues of the standard (N-acetyl cysteine, NAC) compound against venom-related proteins.

Sl. No.	Protein	PDB ID	Binding energy	Key interactions
1	PLA2	1DPY	−4.5	His A: 48, Tyr A: 28, Asp A: 49, Phe A: 101
2	PLA2	1VIP	−4.4	Asp A:49, Cys A:45, Gly A:30
3	PLA2	2PVT	−4.9	Asp A:122, Cys A:27, Cys A:29, Gly A:31
4	PLA2	2PYC	−4.9	Gly A: 32, Gly A: 26, Trp A: 31, Asp A: 122
5	SVSP	3S9B	−4.2	Thr A: 180, Lys A: 230, Ser A: 178
6	SVSP	3SBK	−4	Gly A: 193, Glu A: 218, His A: 192
7	SVMP	2E3X	−5	Ser B: 44, Lys B: 128
8	LAAO	3KVE	−4.8	His D: 223, Gln D: 114, Arg D: 90
9	SNACLEC	3UBU	−4.9	Thr A: 81, Glu A: 82, Trp A: 83, Ser A: 80, Ser B: 71

**FIGURE 1:**
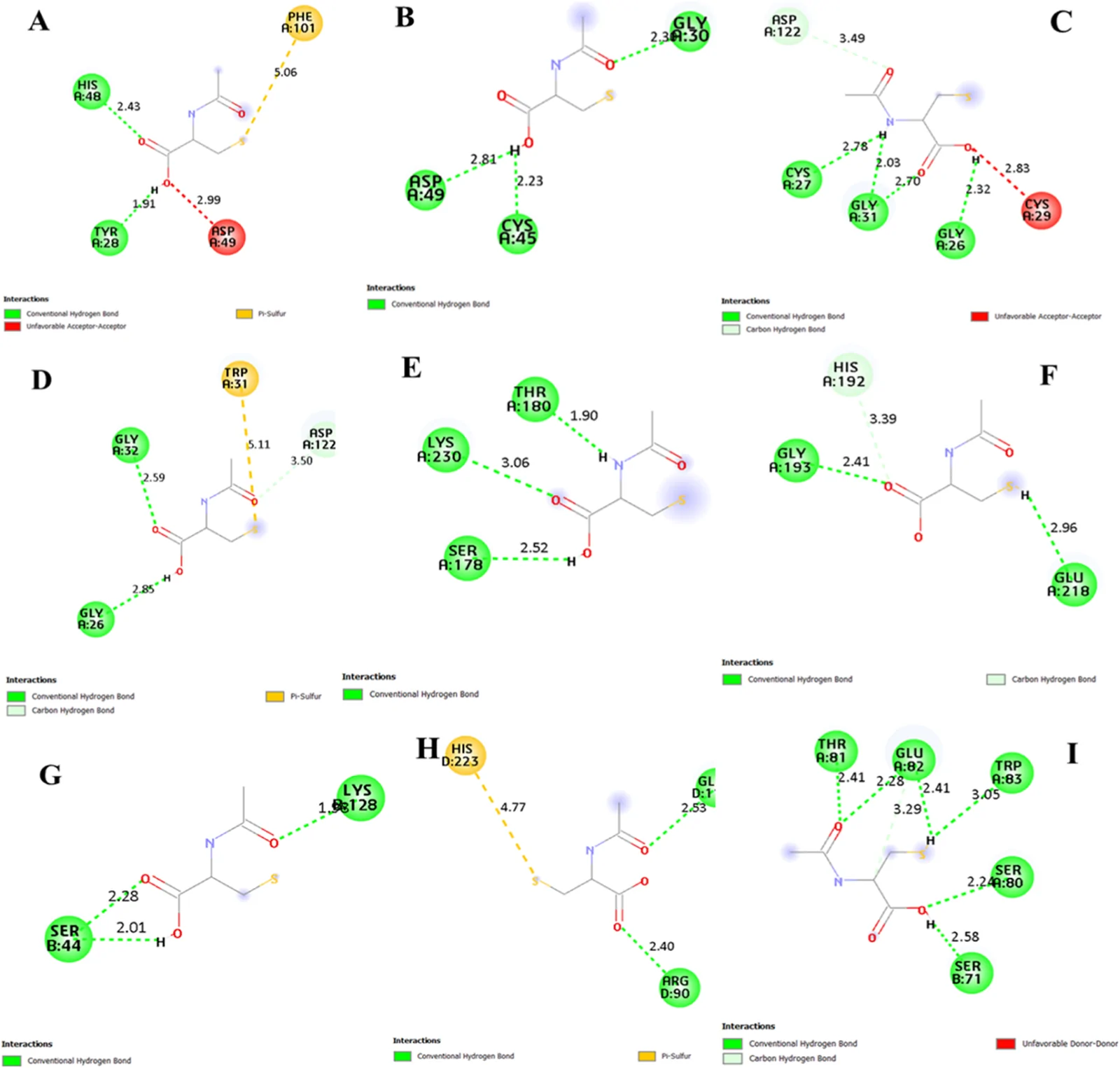
**(A–I)** 2D interaction diagrams of *N*-acetyl cysteine (NAC) with venom-related proteins [**(A)**-1DPY, **(B)**-1VIP, **(C)**-2PVT, **(D)**-2PYC, **(E)**-3S9B, **(F)**-3SBK, **(G)**-2E3X, **(H)**- 3KVE, **(I)**- 3UBU] showing hydrogen bonding, hydrophobic, and other key interactions.

Among the extracts, the methanolic fraction (MME) and petroleum ether fraction (MPE) yielded the most promising hits. The docking scores and 2D interaction diagrams of the best performing compounds are depicted in the Table 2. In particular, 3-chloro-5-cholestene, Vitamin E, and β-sitosterol (from MME and MPE) consistently demonstrated strong binding across phospholipase A_2_ isoforms (1DPY, 1VIP, 2PVT, 2PYC), snake venom serine protease (3S9B), and metalloproteinase (2E3X). These interactions were stabilized by hydrogen bonding, hydrophobic contacts, and π–π stacking with critical catalytic residues such as His48, Asp49, Tyr52, and Phe101 in PLA_2_ and Arg62, Lys73, and Trp173 in SVSP. The petroleum ether fraction further contributed high-affinity ligands such as β-sitosterol and 6-nitro-2-methylpyrrolo [2,3-g] quinoline, which showed potent inhibition potential against SVMP (2E3X) and LAAO (3KVE), respectively. Notably, the best docking scores were reached for β-sitosterol (−9.3 kcal/mol with PLA_2_-1DPY; −8.2 kcal/mol with PLA_2_-1VIP; −8.1 kcal/mol with SVMP-2E3X; −9.3 kcal/mol with LAAO-3KVE), indicating that β-sitosterol has broad inhibitory activity in various venom protein groups. Vitamin E and 3-chloro-5-cholestene were also found to exhibit affinities between −7.0 and −8.9 kcal/mol, supporting them as leading candidates. Based on their binding affinity, toxicological significance, and coverage of major venom protein families, four corresponding protein targets (PLA_2_ (1VIP, 2PYC), SVSP (3S9B), and SVMP (2E3X)) were chosen for 100 ns molecular dynamics (MD) simulations. The top five ligands from MME and MPE from the extracts were identified (β-sitosterol, Vitamin E, 3-chloro-5-cholestene, squalene, and 6-nitro-2-methylpyrrolo [2,3-g] quinoline) for the comparative stability and interaction analysis. NAC was kept for reference to set up a benchmark for the overall inhibitory efficacy of the ligands chosen. Among the nine venom proteins evaluated, four representative proteins (1VIP, 2PYC, 3S9B and 2E3X) demonstrated consistently favourable docking interactions together with high biological relevance and were therefore selected for molecular dynamics simulations.

**TABLE 2:** Docking scores (kcal/mol) and 2D interactions of best-performing compounds of *Glycosmis pentaphylla* against each venom protein based on molecular docking.

Protein	PDB ID	Best compound	Extract	Binding energy (kcal/mol)	2D interactions
PLA_2_	1DPY	β-Sitosterol	MPE	−9.3	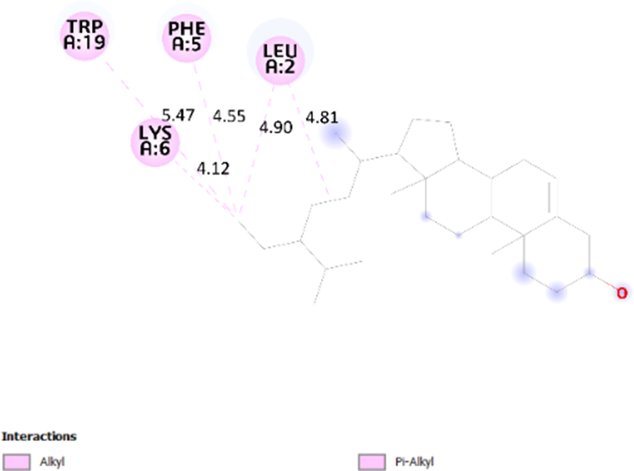
PLA_2_	1VIP	β-Sitosterol	MPE	−8.2	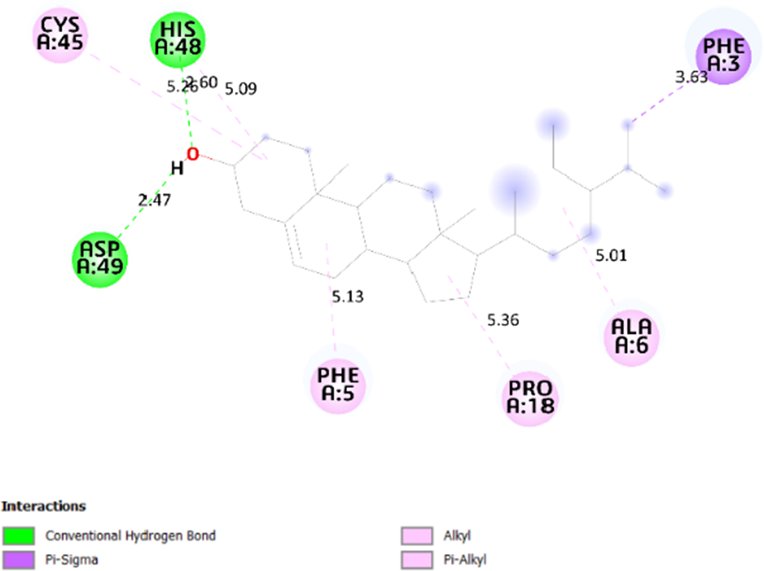
PLA_2_	2PVT	Vitamin E	MME	−7.4	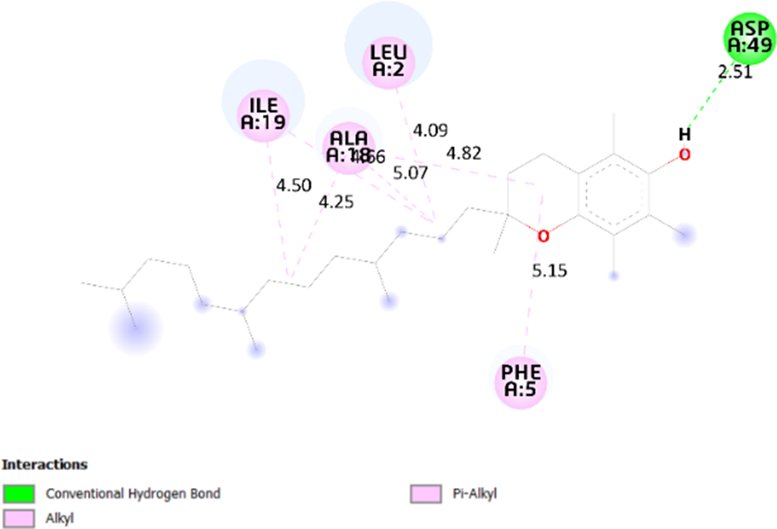
PLA_2_	2PYC	Vitamin E	MME	−8.4	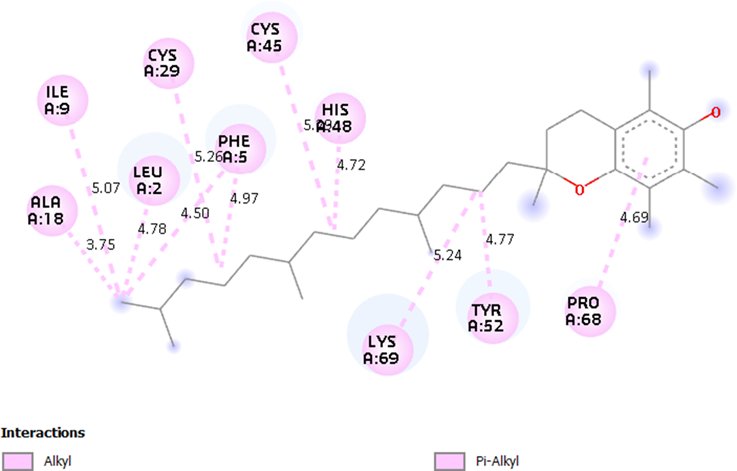
SVSP	3S9B	β-Sitosterol	MPE	−7.6	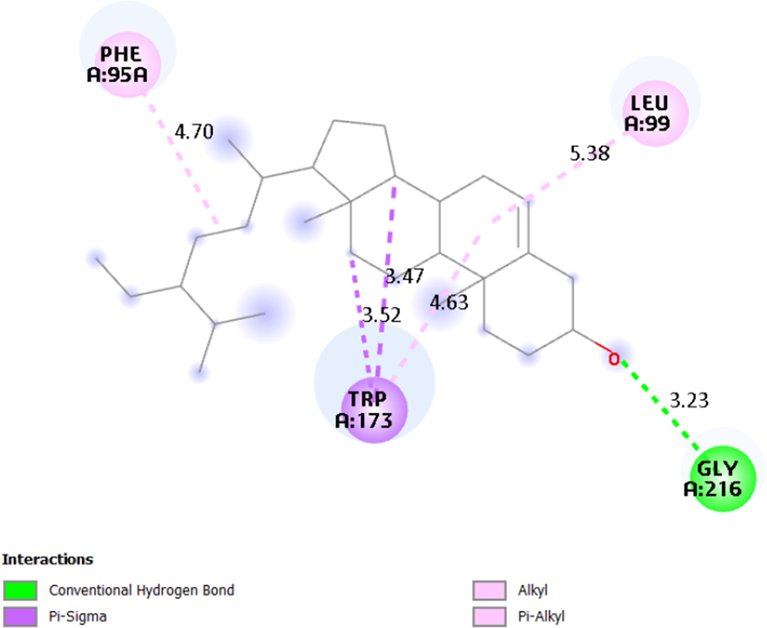
SVSP	3SBK	β-Sitosterol	MPE	−7.0	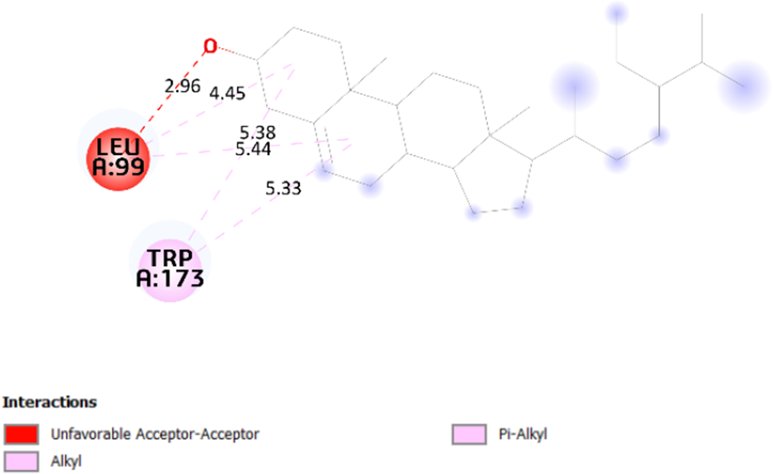
SVMP	2E3X	β-Sitosterol	MPE	−8.1	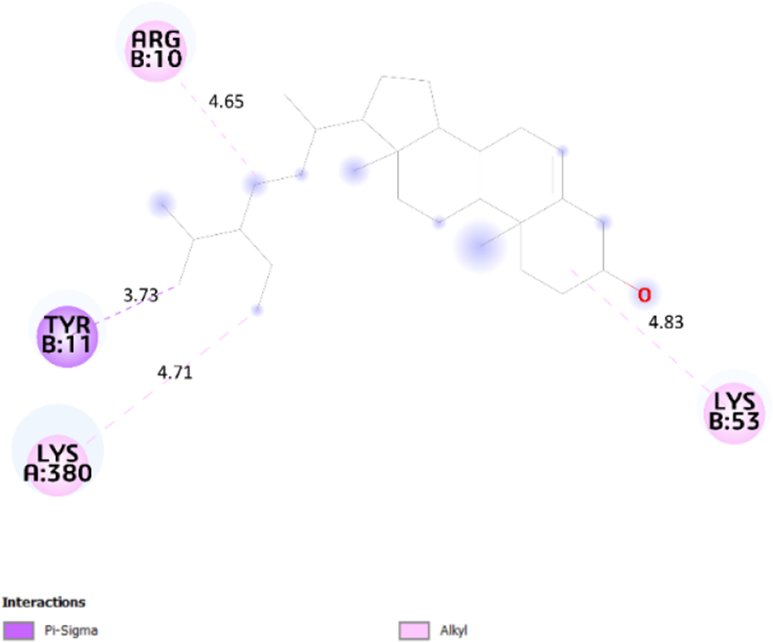
LAAO	3KVE	β-Sitosterol	MPE	−9.3	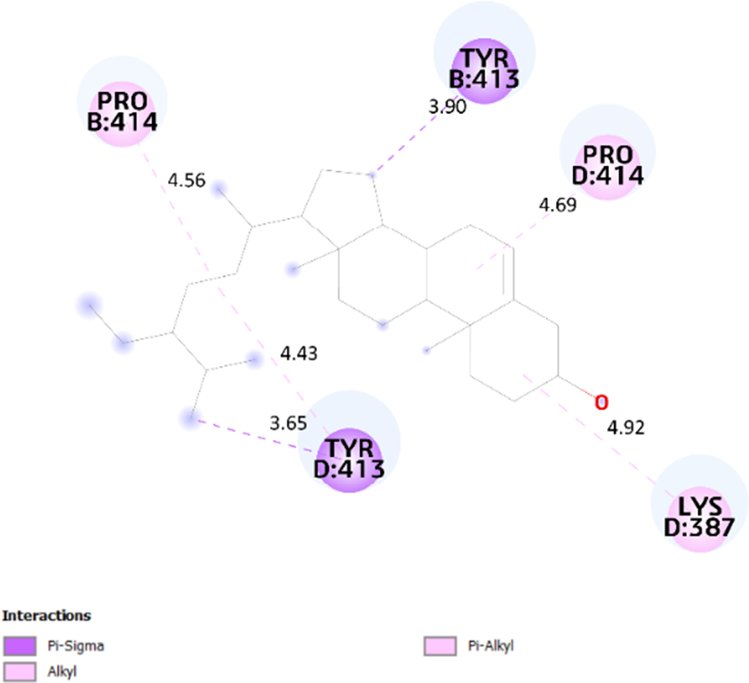
SNACLEC	3UBU	β-Sitosterol	MPE	−6.3	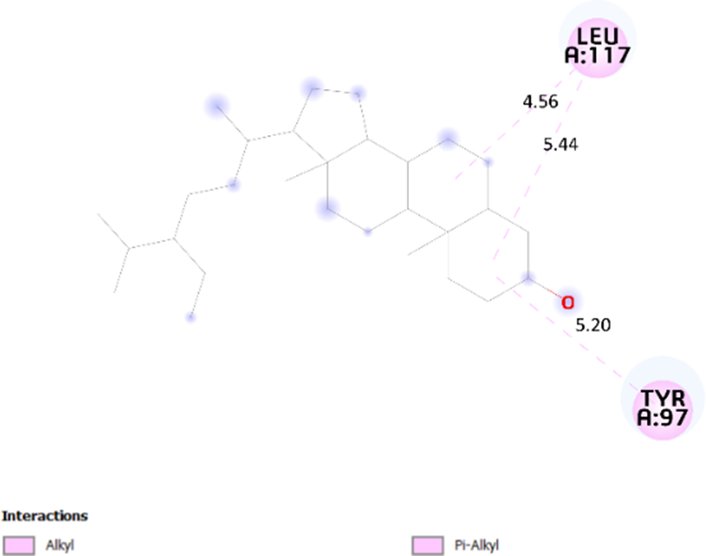

### Molecular dynamics simulation

To further evaluate the stability of the docked protein-ligand complexes, Molecular Dynamics (MD) simulations were carried out on the optimal complexes. Four venom-associated proteins: PLA_2_ (1VIP, 2PYC), SVSP (3S9B), and SVMP (2E3X)-were chosen based on their potent docking affinities, toxicological importance, and structural fit. The top 5 ligands from the methanolic (MME) and petroleum ether (MPE) extracts such as β-sitosterol, Vitamin E, 3-chloro-5-cholestene, squalene, and 6-nitro-2-methylpyrrolo [2,3-g] quinoline, were compared with 100 ns simulations and N-acetylcysteine (NAC) as the reference compound. This approach enabled the characterization of conformational stability, residue flexibility, compactness, and binding endurance when occurring under physiologically significant conditions. The simulation path was evaluated by five dynamic descriptors, which included Root Mean Square Deviation (RMSD), Root Mean Square Fluctuation (RMSF), Radius of Gyration (Rg), Hydrogen Bonding (H-bonds), and Solvent Accessible Surface Area (SASA), leading to a comparative characterization of complex stability, ligand affinity and interaction dynamics.

### RMSD analysis: global conformational stability of protein-ligand complexes

Root Mean Square Deviation (RMSD) was employed to evaluate the global conformational stability of the four venom-associated proteins (1VIP, 3S9B, 2E3X, and 2PYC) in complex with the top-ranked selected ligands and the reference compound NAC during the 100 ns MD simulations. In all systems, RMSD profiles stabilized after the initial equilibration phase (10–15 ns), indicating well-equilibrated dynamics. The RMSD trajectories of the protein–ligand complexes (1VIP, 3S9B, 2E3X, and 2PYC) with the top selected ligands and NAC are presented in Figures 2A–D, illustrating the comparative conformational stability of each system over the 100 ns simulation.

**FIGURE 2:**
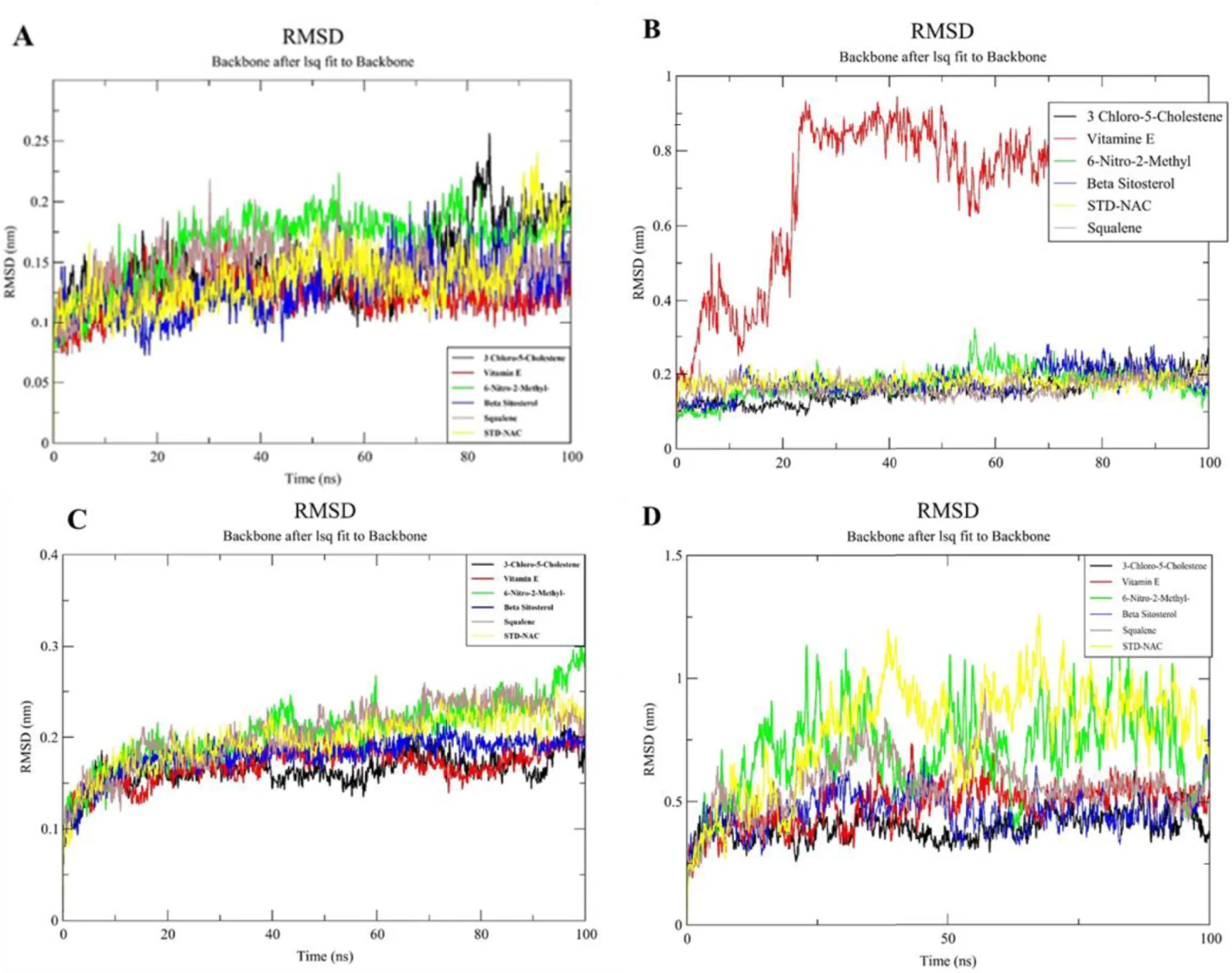
RMSD profile of **(A)** 1VIP, **(B)** 2PYC, **(C)** 3S9B and **(D)** 2E3X in complex with selected ligands and NAC over 100 ns simulation.

For PLA_2_ (1VIP), NAC exhibited moderate stability with RMSD values ranging between 0.21–0.26 nm. Of the selected ligands, 3-chloro-5-cholestene (L1) showed the highest stability by preserving a small RMSD range of 0.15–0.18 nm, whereas Vitamin E (L2) (0.18–0.21 nm). Contrarily, Squalene (L5) displayed larger deviations (≈0.24–0.27 nm)-indicating poorer stability of the protein backbone. For SVSP (3S9B), NAC kept RMSD values between 0.23–0.26 nm. With 3-Chloro-5-cholestene, the most stable complex was presented, such that no deviations beyond 0.17–0.20 nm were observed, which implied minimal conformational drift. Ligands 2 and 3 showed moderate stability (0.20–0.23 nm), whereas selected ligands 4 and 5 exhibited RMSD spikes above 0.25 nm occasionally representing ligand reorientation or partial shift from the active site. As is the case in SVMP (2E3X), NAC exhibited moderate variations (0.23–0.26 nm). While 3-chloro-5-cholestene was again the most stable trajectory, exhibiting RMSD range of 0.17–0.20 nm. Similarly, Vitamin E (L2) was stable (0.19–0.22 nm), whereas Squalene (L5) exhibited large deviations (∼0.24–0.27 nm), demonstrating limited capacity to moderate the conformational movements of the protein. In PLA_2_ (2PYC), the NAC complex was found to exhibit oscillations of 0.20–0.35 nm, consistent with moderate stability. Out of the ligands selected, Ligands 3 and 5 demonstrated greater stability in trajectory overall compared with NAC (0.15–0.25 nm), which indicated that their binding was stable. By comparison, for Ligand 1, the deviations were higher (∼0.40 nm) after 60 ns, indicating higher conformational flexibility or lower binding capacity. In general, RMSD analysis showed that the selectively chosen ligands-3-chloro-5-cholestene, Vitamin E, and Ligand 3 specifically-produced better structural stabilization of venom proteins than NAC. This indicates their potential to successfully confine protein flexibility, which is requisite for the neutralization of enzymatic and toxic activities of viper venom proteins.

### Residue-level fluctuation and catalytic site stabilization at RMSF

The residue-level displacement of each of the four venom complexes (in association with selected ligands and NAC) during the 100 ns simulations was analyzed for RMSF (Figures 3A–D). In general, terminal regions were more mobile as anticipated for solvent exposed loops, with ligand-dependent effects more prominent near catalytically significant residues and protein-recognition pockets.

**FIGURE 3:**
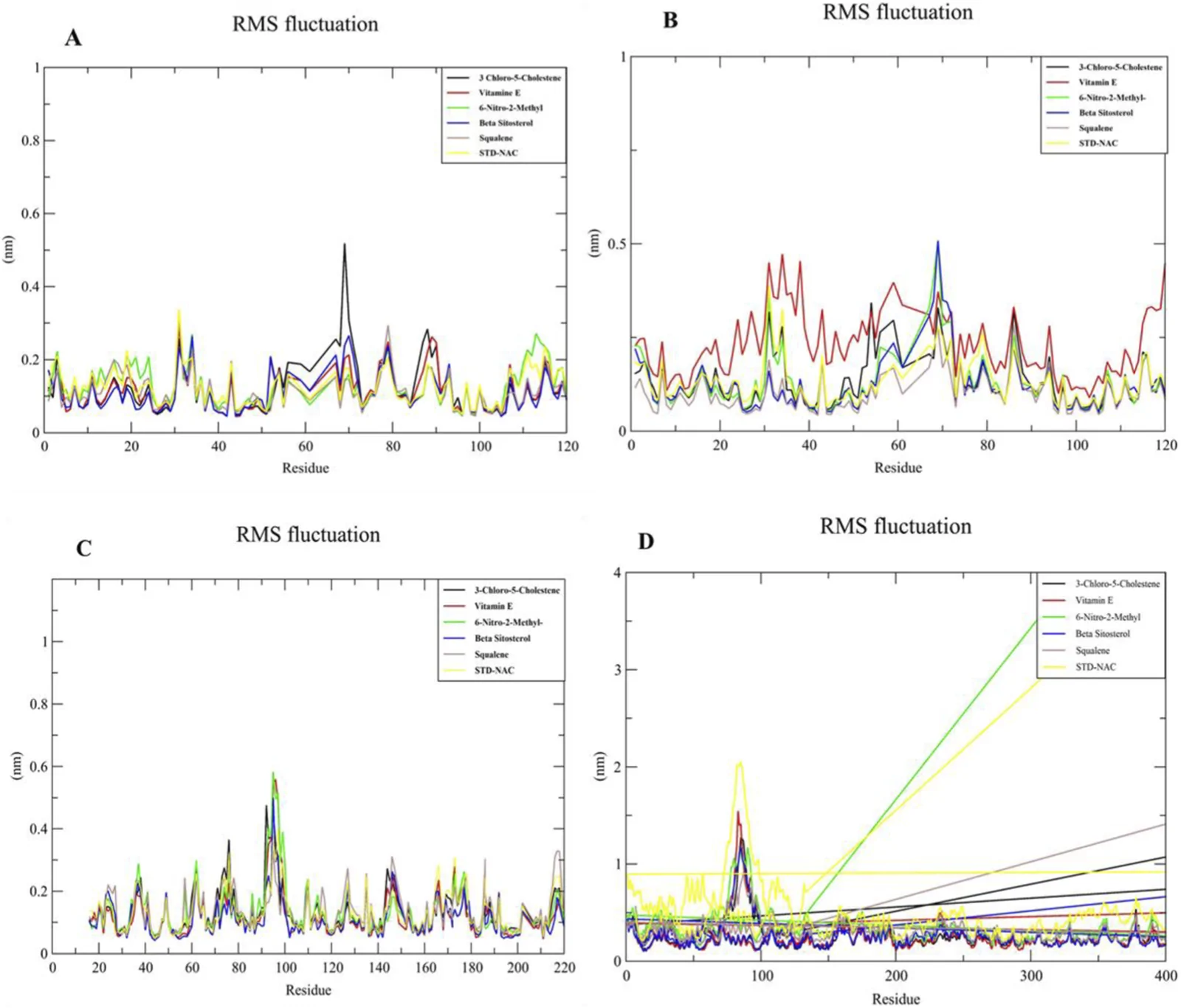
RMSF plots of **(A)** 1VIP, **(B)** 2PYC, **(C)** 3S9B and **(D)** 2E3X showing residue-specific fluctuations across complexes.

In PLA_2_ (1VIP), high variability was found in the NAC complex surrounding the catalytic dyad (His48, Asp49, and Tyr52) with an average RMSF larger than 0.12 nm. However, fluctuations in these residues at ∼0.08–0.10 nm were noticeably diminished by 3-chloro-5-cholestene (L1) and Vitamin E (L2), which showed an increase in the stability of the active site. Other ligands (L3-L5) exhibited more flexibility (∼0.11–0.13 nm), especially in the loop and terminal regions, indicating limited structural restraint. In SVSP (3S9B) NAC enabled high flexibility in loop regions (residues 40–80 and 180–210) including the catalytic triad (His57, Asp102, Ser195) and up to 0.15 nm peaks. Ligand 1 also strongly suppressed the fluctuations in the active site loop (residues 55–65), leading to values of 0.08–0.10 nm. Ligand 2 exerted intermediate stability of moderate stability, whereas ligands 3–5 exhibited some loss of flexibility, but were not fully inhibiting the movement of the catalytic site. For SVMP (2E3X), the NAC complex showed higher level of mobility of catalytic zinc-binding residues (His142, Glu143, His146), and variations averaged 0.12–0.14 nm. Additionally, 3-chloro-5-cholestene (L1) successfully prevented such deviations (∼0.08–0.10 nm) and Vitamin E (L2) significantly reduced loop flexibility compared with NAC. Stabilization indicates that, in particular, L1 restricts structural rearrangements necessary for substrate access and zinc-mediated catalysis. In PLA_2_ (2PYC), the majority of the residues showed fluctuations between 0.10–0.25 nm. NAC provided moderate flexibility of loop binding sites, whereas Ligands 2 and 3 substantially decreased fluctuations at residues 55–80 and 140–160-highlighting enhanced stabilization on binding sites. Ligand 4, in contrast, led to localized enhanced flexibility that reflects weaker or transient interactions. Together, RMSF-rated ligands, especially 3-chloro-5-cholestene, Vitamin E, and Ligand 3, provided significantly greater local stabilization of the catalytic residues than NAC. It would therefore be expected to exhibit residue-level rigidity, in line with successful functional inhibition, as less movement in active sites is essential for minimizing the enzymatic activity of venom proteins.

### Radius of gyration (Rg): structural compactness of protein-ligand complexes

The radius of gyration (Rg) of protein-ligand complexes was analysed for global compactness and to assess the stability of folding in these protein–ligand complex architectures over 100 ns simulations. In all systems, the Rg values of NAC were not significantly different from expected (∼1.87–1.91 nm), indicating moderate structural compactness. However, binding of specific ligands varied the effect on the protein folding, as many ligands were found to result in stronger binding for protein folding than NAC, but all ligands showed different results. The Rg trajectories of all protein-ligand complexes (1VIP, 2PYC, 3S9B and 2E3X) on the 100 ns simulations are depicted in Figures 4A–D, demonstrating that NAC and specific ligands have their own individual advantages for different protein compactness.

**FIGURE 4:**
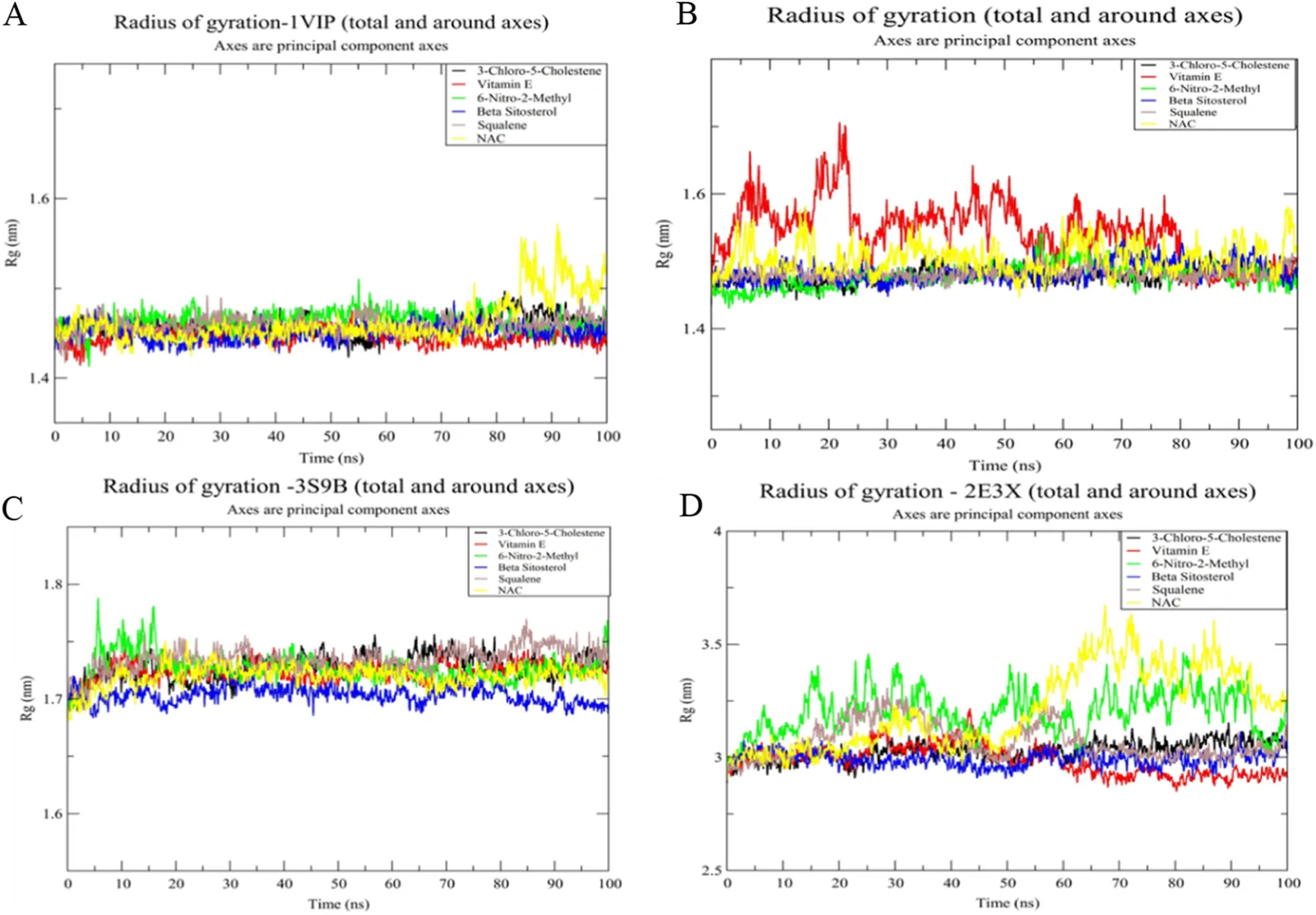
Radius of gyration profiles for **(A)** 1VIP, **(B)** 2PYC, **(C)** 3S9B and **(D)** 2E3X -ligand complexes showing structural compactness.

For PLA_2_ (1VIP), the compact-defined conformation of the L1 complex (3-chloro-5-cholestene) is more observed with Rg values remaining within 1.78–1.81 nm at different points and indicates ligand induced stiffening that could prevent the substrate from being accessed. Vitamin E (L2) and other mediators retained intermediate compactness (1.82–1.86 nm), while Squalene (L5) presented the softest packing (1.86–1.90 nm), which is in accordance with its high RMSD and SASA values. For PLA_2_ (2PYC), the NAC complex was kept at the peak close to 1.88–1.90 nm. Ligands 3 and 5 complexes had slightly small Rg values (∼1.85–1.88 nm), which is reflected in its enhanced structural compactness, whereas Ligands 1 and 4 reported small deviations, indicating their local unfolding tendency and lower stabilizing potential. For SVSP (3S9B), the L1 complex also exhibited high compactness, with the Rg value of 3S9B L1, that is in this range of 1.79–1.82 nm, compared to 1.87–1.90 nm for NAC. The stability values of ligands 2 and 3 also corresponded to 1.83–1.85 nm, whereas those of Ligands 4 and 5 exhibited more variability beyond 70 ns, showing lower stability. Compared to SVMP (2E3X), NAC gave a relaxed (1.88–1.91 nm) profile. The Rg values (1.79–1.82 nm) were the lowest for all ligands, showed by the bond of 3-chloro-5-cholestene (L1) indicating tight and thermodynamically stable arrangement. Vitamin E (L2) enhanced compactness of ∼1.80–1.83 nm, while Squalene (L5) increased as expected over time, indicating an inappropriate stability. Reversal of some ligands was found to promote structural compactness of venom proteins, specifically 3-chloro-5-cholestene or Vitamin E, in contrast to NAC, confirming that these ligands provided conformational stability and limited the enzymatic activity.

### Hydrogen-bonding analysis: strength and specificity of hydrogen bonding analysis

Hydrogen bond properties were analysed in order to analyze the size, persistence, and functional relevance of ligand-protein conjugates between the four venom related proteins. Generally, only 1–2 transient hydrogen bonds were present throughout the complexes as a typical hydrogen bond in NAC and resulted in moderate binding affinity and relatively poor binding ability in the active sites. In contrast, the hydrogen bonding strength and persistence of some of the chosen ligands are significantly improved, which favours stronger binding and inhibition stability. Hydrogen bond profiles of the four protein-ligand complexes using standard NAC are shown in Figures 5A–D.

**FIGURE 5:**
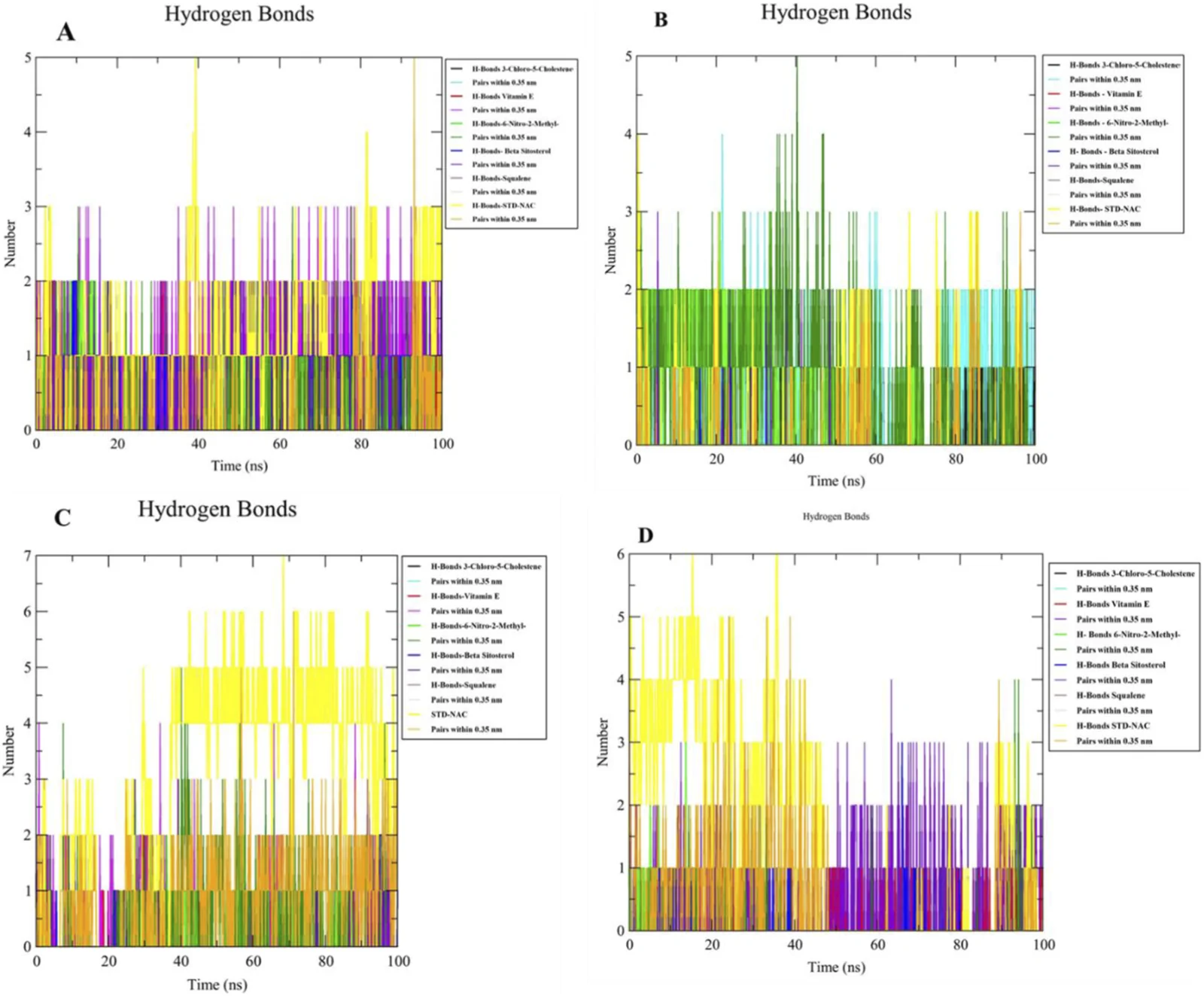
**(A–D)** Hydrogen bonding dynamics of selected ligands and NAC with **(A)** 1VIP, **(B)** 2PYC, **(C)** 3S9B and **(D)** 2E3X during simulation.

In PLA_2_ (1VIP), L1 consistently formed 3–5 hydrogen bonds from its catalytically important residues (Ser29, Gly30 and His48). Vitamin E (L2) also exhibited good bonding behaviour (3–4 hydrogen bonds) but other ligands (L3-L5) formed fewer and less persistent interactions. Remarkably, Squalene (L5) showed no interaction with the catalytic residues, in line with weak binding overall. In PLA_2_ (2PYC) the NAC produced only 1–2 transient hydrogen bonds. In particular, Ligand 3 displayed the strongest, most consistent (3–4 hydrogen bonds throughout the simulation), followed by Ligands 2 and 5, exhibiting stable interactions with important binding site residues. Ligand 1 exhibited unstable hydrogen bonding characteristics which reflected poorly on the binding dynamics by regularly breaking and reforming hydrogen bonds. In SVSP (3S9B), NAC once again revealed few interactions, forming only 1–2 hydrogen bonds with peripheral residues. The interaction profile of selected ligand 1 was more robust, forming between 4 and 5 stable hydrogen bonds throughout the simulation, especially with catalytically relevant residues such as Ser195 and Gly193. Selected ligands 4 and 5 exhibited irregular and unstable binding trends whereas selected ligands 2 and 3 maintained persistent hydrogen bonds (2–3).

According to SVMP (2E3X), hydrogen bonding was essential for binding ligands in the catalytic zinc binding region. NAC exhibited only 1–2 weak and transient hydrogen bonds whereas L1 (3-chloro-5-cholestene), consistently formed 4–5 hydrogen bonds with active site residues such as Glu143, Tyr145, and His146, confirming its high binding capacities. Vitamin E (L2) also indicated stable hydrogen bonds (3–4 bonds), and L5 showed erratic and transient interactions. On a whole, the hydrogen bond analysis revealed that 3-chloro-5-cholestene, Vitamin E and Ligand 3 showed a higher capability of hydrogen bonding than NAC and other ligands. Its engagement of catalytic residues by enduring hydrogen bonding likely contributes towards high binding orientation, structural stability and almost certainly action against enzyme activities necessary for venom toxicity.

### Solvent accessible surface area (SASA): width of attachment and solvent disguise

The Solvent Accessible Surface Area (SASA) was used to measure ligand encapsulation and solvent exposure of the protein interface during the 100 ns MD simulations. Ligand burial in the binding cleft, limited solvent access of the catalytic residues, and improved stability of the protein-ligand complexes usually led to lower SASA values. The SASA characteristics of the protein-ligand complexes are given in Figures 6A–D.

**FIGURE 6:**
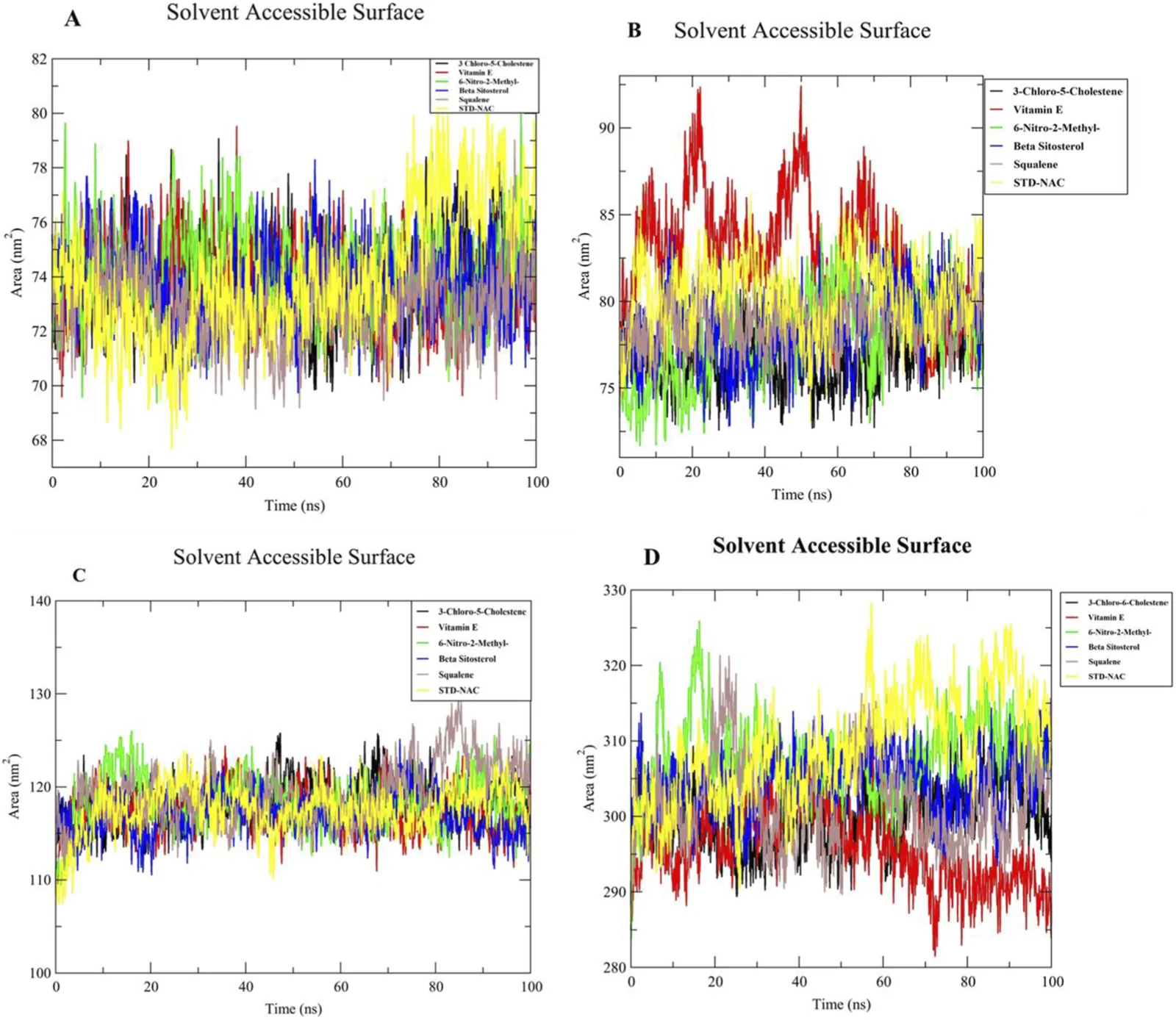
**(A–D)** SASA analysis of selected ligands and NAC with **(A)** 1VIP, **(B)** 2PYC, **(C)** 3S9B and **(D)** 2E3X complexes reflecting ligand depth and solvent shielding.

SASA values of NAC complex in PLA_2_ (1VIP) achieved up to ∼125 nm^2^ with superficial interaction. However, 3-chloro-5-cholestene (L1) was able to reduce the SASA to 112–115 nm^2^, indicating deep binding site insertion with a good protected catalytic pocket. Vitamin E (L2) showed modest SASA reduction (116–118 nm^2^) while Squalene (L5) yielded its peak (∼126 nm^2^), indicating weak binding and poor burrow into the hydrophobic cleft. In PLA_2_ (2PYC), the SASA value for NAC was 125–130 nm^2^. And the same reductions were observed for Ligand 3 and Ligand 5 (between ∼120 and 125 nm^2^–higher packing, lower solvent contact). However, Ligand 1 increased SASA to approximately 135 nm^2^, aligned with the higher conformational fluctuations measured in RMSD and RMSF analysis and suggesting a less favorable binding orientation. NAC for SVSP (3S9B) kept SASA values around 130 nm^2^ suggesting minimal insertion. However, phyto ligand 1 also resulted in a remarkable decrease to 112–115 nm^2^, indicating increased burial and shielding of functional residues. Specially chosen ligands (2–5) induced intermediate reductions in SASA, in contrast to the burial depth observed for selected ligand 1, and thus, further reinforcing its better stabilization response. The SASA for NAC complexes (125–128 nm^2^) from SVMP (2E3X) was very low. This L1 complex gave the lowest SASA of about 112–115 nm^2^, reflecting the deep embedding in the catalytic cleft and also lower solvent accessibility to the zinc-coordinating residues. Vitamin E (L2) showed intermediate burial (116–118 nm^2^) while L5 was surface-oriented (∼126–128 nm^2^) indicating a weak interaction profile. SASA showed that 3-chloro-5-cholestene (L1) had the most pronounced binding for different venom proteins, and was succeeded by Vitamin E (L2) and Ligand 3, whereas NAC and less strong ligands showed superficial binding. Finally, based on the RMSD and RMSF as well as Rg data, L1 was identified to be the most promising inhibitor, and is shown to stabilize protein conformation, providing sterically resistant sites to prevent the enzyme from escaping the solvent.

To further evaluate the therapeutic relevance of the selected phytocompounds, pharmacokinetic profiling, toxicity prediction, and pathway enrichment analyses were performed. The top-performing ligands identified from docking and molecular dynamics simulations were subjected to SwissADME and ProTox-II analyses, followed by STRING-based protein-protein interaction and pathway enrichment studies to explore the biological mechanisms associated with venom neutralization.

### Biological validation of protease inhibitory activity using a trypsin-casein model system

To experimentally validate the protease-targeting potential predicted by molecular docking and molecular dynamics simulations, the protease inhibitory activity of *Glycosmis pentaphylla* extract was evaluated using both a trypsin-casein digestion assay and a skim milk agar plate assay. Trypsin was employed as a model serine protease due to its functional similarity to proteolytic enzymes present in snake venoms. The ability of the extract to suppress trypsin-mediated proteolysis was considered preliminary evidence supporting the *in silico* prediction of protease inhibition.

### Trypsin-casein protease inhibition assay

The extract exhibited concentration-dependent inhibition of trypsin-mediated casein hydrolysis. The absorbance of the positive control (trypsin + casein) was 0.2373 ± 0.0034, whereas treatment with *G. pentaphylla* extract progressively reduced protease activity and also depicted in Table 3; Figures 7, 8. The percentage inhibition increased from 19.61% at 1 mg/mL to 56.86% at 5 mg/mL. One-way ANOVA revealed a highly significant difference among treatment groups (F = 191.8, P < 0.0001). Dunnett’s multiple comparison test demonstrated that all extract concentrations significantly reduced protease activity compared with the positive control (P < 0.0001). These findings indicate the presence of bioactive constituents capable of interfering with proteolytic enzyme activity and provide experimental support for the protease-targeting interactions predicted by molecular docking and molecular dynamics simulations.

**TABLE 3:** Concentration-dependent inhibition of trypsin-mediated casein hydrolysis by *Glycosmis pentaphylla* leaf extract.

Treatment groups	Mean (OD 595)	SD	Mean ± SD	% of inhibition
Positive control	0.23725	0.003403	0.2373 ± 0.0034	-
S1 (1 mg/mL))	0.21475	0.004349	0.2148 ± 0.0043	19.60784314
S2 (2 mg/mL))	0.2045	0.003109	0.2045 ± 0.0031	28.54030501
S3 (3 mg/mL))	0.19875	0.003096	0.1988 ± 0.0031	33.55119826
S4 (4 mg/mL))	0.18425	0.003403	0.1843 ± 0.0034	46.18736383
S5 (5 mg/mL))	0.172	0.00216	0.1720 ± 0.0022	56.8627451
Negative control	0.1225	0.002646	0.1225 ± 0.0026	-

**FIGURE 7:**
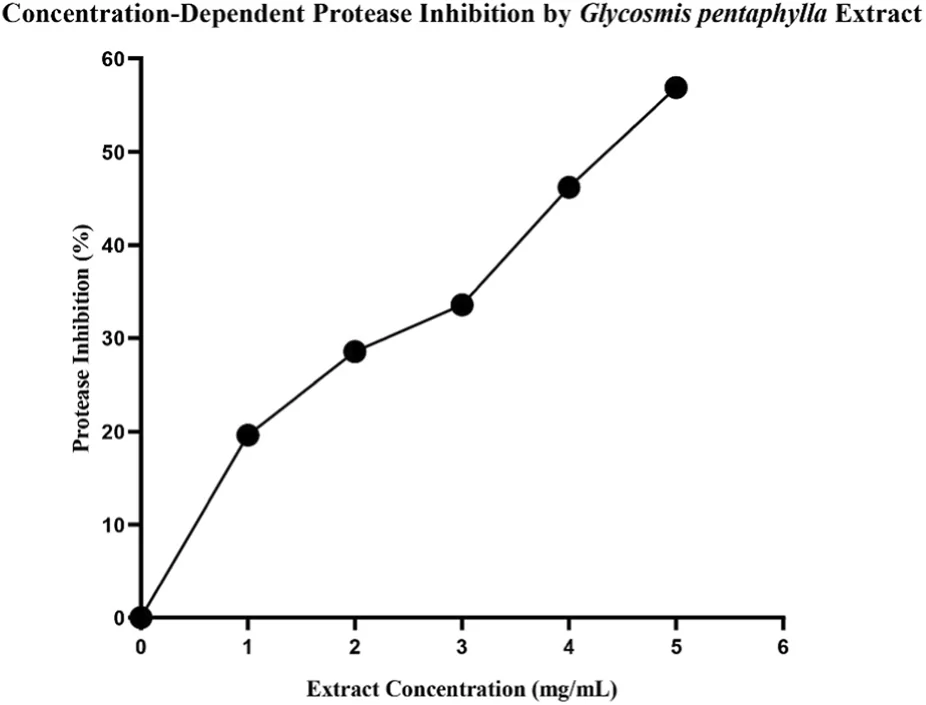
Protease inhibitory activity of *Glycosmis pentaphylla* extract against trypsin-mediated casein digestion.

**FIGURE 8:**
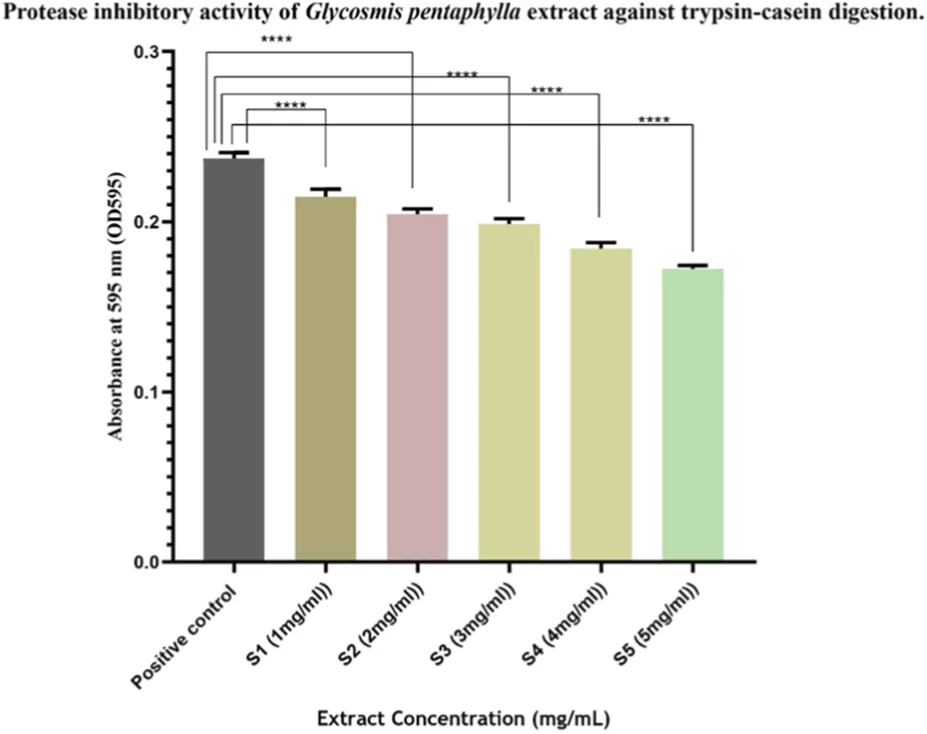
Protease inhibitory activity of *Glycosmis pentaphylla* extract against trypsin-mediated casein digestion. Data are presented as mean ± SD (n = 4). Statistical analysis was performed using one-way ANOVA followed by Dunnett’s multiple comparison test. Significant differences compared with the positive control are indicated by **** (P < 0.0001).

### Skim milk agar protease inhibition assay

The protease inhibitory effect of *Glycosmis pentaphylla* extract was further confirmed using a skim milk agar plate assay. The caseinolytic zone of trypsin alone was 17 mm, suggestive of proteolysis. By contrast, the use of trypsin with extract resulted in a diameter of zone of 12 mm, indicating a protease inhibition of 28.19 percent. There were no clearance zones in either the extract control or negative control, confirming that the extract itself had not hydrolyzed casein. With a decrease in the hydrolysis region, the extract demonstrates the reduction of the protease-mediated degradation of the substrate and has also been visually confirmed for the action inhibition of the liquid-phase method. Inhibition area is presented in Table 4 and skim milk plate is in Figure 9.

**TABLE 4:** Inhibitory effect of *Glycosmis pentaphylla* extract on trypsin-induced casein hydrolysis in the skim milk agar assay.

Group	Zone of clearance (mm)	Mean ± SD
Positive control (trypsin)	17 mm,17.5mm, 17 mm	17.17 ± 0.29
Test (trypsin + extract)	12 mm, 12mm, 13 mm	12.33 ± 0.58
Extract control	0 mm	-
Negative control (water)	0 mm	-

**FIGURE 9:**
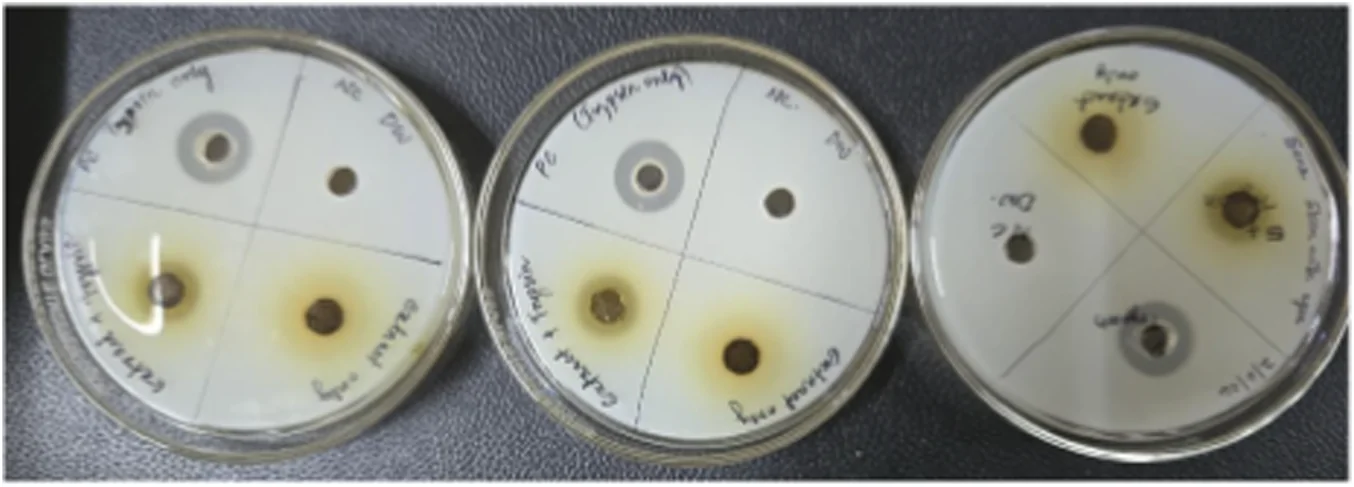
Representative triplicate skim milk agar plates showing protease inhibitory activity of *Glycosmis pentaphylla* extract against trypsin.

### % Protease inhibition

$$\%\text{Inhibition}=\frac{17.17-12.33}{17.17}\times 100=28.19\%$$

The positive control (trypsin) exhibited a prominent caseinolytic zone, whereas the test group (trypsin + extract) showed a reduced zone of clearance. No hydrolysis zones were observed in the extract control or negative control, confirming the absence of intrinsic proteolytic activity.

### Biological validation of membrane stabilizing activity using an osmotic stress-induced erythrocyte hemolysis model

#### Membrane stabilizing activity

The membrane stabilizing potential of *Glycosmis pentaphylla* extract was evaluated using an osmotic stress-induced erythrocyte hemolysis model. Exposure of red blood cells to hypotonic conditions resulted in substantial hemolysis in the positive control group (OD540 = 0.249 ± 0.004), whereas treatment with the extract significantly reduced hemoglobin release in a concentration-dependent manner. The absorbance progressively decreased from 0.211 ± 0.002 at 1 mg/mL to 0.171 ± 0.001 at 5 mg/mL, corresponding to membrane stabilization values of 19.19% and 39.23%, respectively. One-way ANOVA demonstrated highly significant differences among the treatment groups (F = 409.5, P < 0.0001), while Dunnett’s multiple comparison test confirmed significant protection at all tested concentrations compared with the positive control. These findings indicate that the extract possesses appreciable membrane protective activity and may contribute to mitigating venom-associated haemolytic damage through stabilization of cellular membranes. The % membrane stabilization activity was shown in the Table 5 and Figures 10, 11.

**TABLE 5:** Membrane stabilizing activity of *Glycosmis pentaphylla* extract against osmotic stress-induced erythrocyte hemolysis.

Treatments	Mean	SD	OD540 (Mean ± SD	% Membrane stabilization
Positive control	0.248667	0.003512	0.249 ± 0035	-
Negative control	0.050667	0.002082	0.051 ± 0020	-
S1 (1 mg/mL)	0.210667	0.002082	0.211 ± 0020	19.19191919
S2 (2 mg/mL)	0.200667	0.001528	0.201 ± 0016	24.24242424
S3 (3 mg/mL)	0.187	0.003606	0.187 ± 0036	31.14478114
S4 (4 mg/mL)	0.179667	0.001155	0.179 ± 0012	34.84848485
S5 (5 mg/mL)	0.171	0.001	0.171 ± 001	39.22558923

**FIGURE 10:**
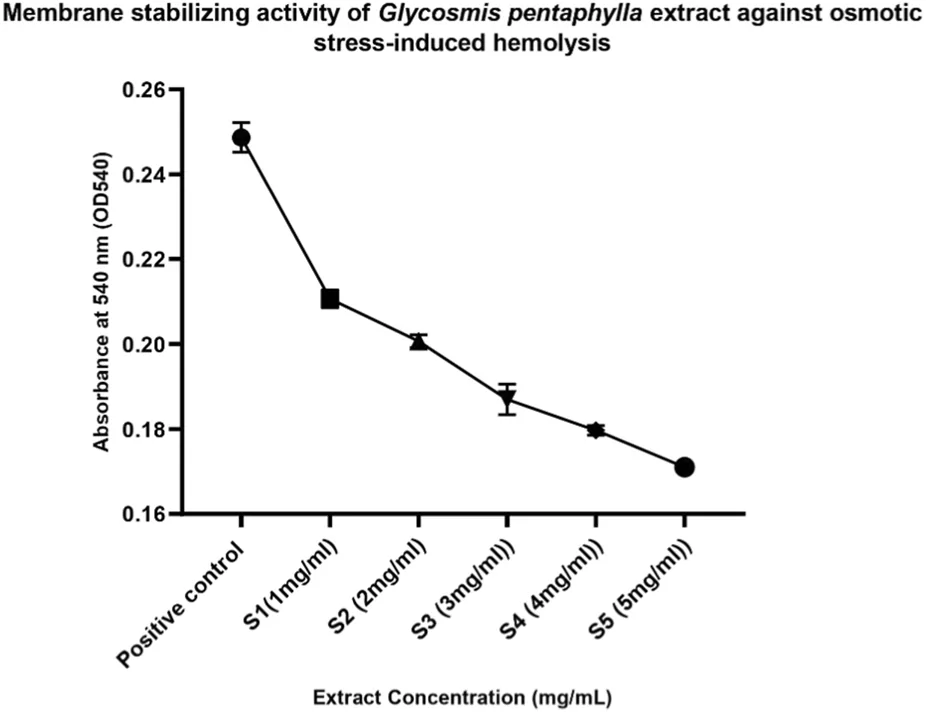
Membrane stabilizing activity of *Glycosmis pentaphylla* extract against osmotic stress-induced hemolysis. Data are expressed as mean ± SD (n = 3). Statistical significance was determined using one-way ANOVA followed by Dunnett’s multiple comparison test.

**FIGURE 11:**
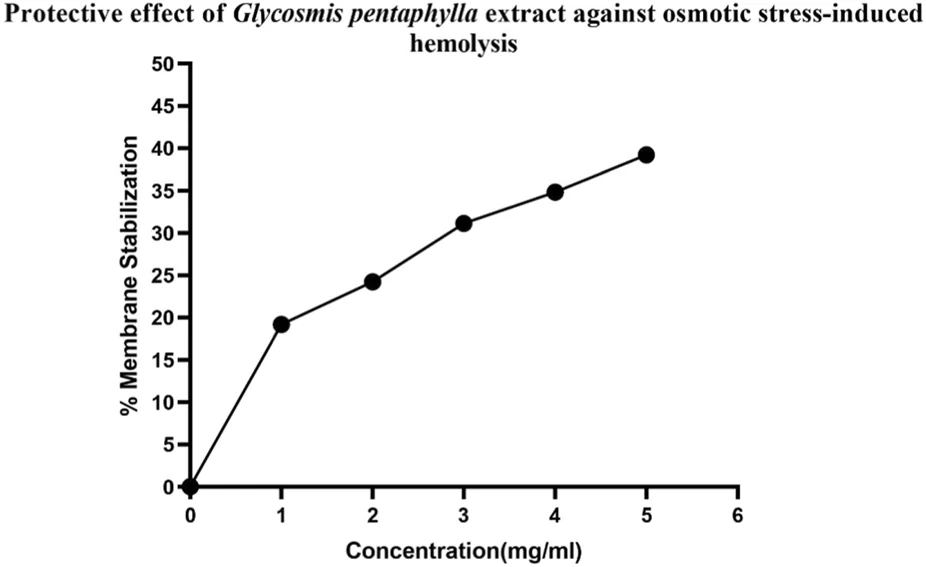
Concentration-dependent membrane stabilization by *Glycosmis pentaphylla* extract against osmotic stress-induced erythrocyte hemolysis. Maximum membrane stabilization (39.23%) was observed at 5 mg/mL.

#### Pharmacokinetic and drug-likeness analysis

The pharmacokinetic properties of the selected phytocompounds were evaluated using SwissADME (Table 6). β-sitosterol, vitamin E, 3-chloro-5-cholestene, and squalene showed high lipophilicity, with LogP values ranging from 8.02 to 10.60, whereas 6-nitro-2-methylpyrrolo [2,3-g] quinoline and NAC exhibited comparatively lower LogP values. All compounds demonstrated acceptable bioavailability scores (∼0.55) and also it is depicted in the Table 6.

**TABLE 6:** Pharmacokinetic and drug-likeness properties of selected phytocompounds predicted using SwissADME.

Parameters	3-Chloro-5-cholestene	Vitamin E	β-sitosterol	Squalene	6-Nitro-2-methylpyrrolo [2,3-g] quinoline	N- Acetyl cysteine
Molecular weight	405.1	430.71	414.71	410.72	227.22	163.2
LogP (WLOGP)	8.64	8.84	8.02	10.60	2.93	−0.49
H-bond donors/acceptors	0/0	1/2	1/1	0/0	1/3	2/3
GI absorption	Low	Low	Low	Low	High	High
BBB permeability	No	No	No	No	No	No
Lipinski violations	Yes; 1 violation: MLOGP>4.15	Yes; 1 violation: MLOGP>4.15	Yes; 1 violation: MLOGP>4.15	Yes; 1 violation: MLOGP>4.15	Yes; 0 violation	Yes; 0 violation
Bioavailability score	0.55	0.55	0.55	0.55	0.55	0.56
Water solubility	0.45 (1.14e+03 mg/mL; 2.81e+00 mol/L)- Highly soluble	−0.17 (2.91e+02 mg/mL; 6.76e-01 mol/L) Very soluble	0.02 (4.38e+02 mg/mL; 1.06e+00 mol/L) Highly soluble	0.45 (1.15e+03 mg/mL; 2.81e+00 mol/L)- Highly soluble	−1.12 (1.74e+01 mg/mL; 7.66e-02 mol/L) Very soluble	−1.76 (2.83e+00 mg/mL; 1.74e-02 mol/L) Very soluble

Most sterol- and terpenoid-based compounds displayed a single Lipinski violation associated with elevated lipophilicity. In contrast, NAC and 6-nitro-2-methylpyrrolo [2,3-g] quinoline complied fully with Lipinski’s rule of five and showed high gastrointestinal absorption. None of the selected compounds were predicted to cross the blood-brain barrier. Water solubility varied among the compounds, with NAC and 6-nitro-2-methylpyrrolo [2,3-g] quinoline showing comparatively better solubility profiles. Overall, the selected phytocompounds demonstrated acceptable pharmacokinetic characteristics suitable for further computational evaluation. Although β-sitosterol, vitamin E and squalene demonstrated favourable binding affinities, their high lipophilicity and poor aqueous solubility may restrict systemic bioavailability. Consequently, formulation approaches such as nanoparticle encapsulation or lipid-based delivery systems may be required to improve therapeutic applicability.

#### Toxicity prediction analysis

The toxicity profiles of the selected phytocompounds were predicted using the ProTox-II platform (Table 7). Most compounds were classified within toxicity Classes IV and V, indicating relatively low predicted acute oral toxicity. Vitamin E and squalene showed the highest predicted safety with LD50 values of 5,000 mg/kg, while β-sitosterol and 3-chloro-5-cholestene exhibited moderate toxicity profiles.

**TABLE 7:** In silico toxicity assessment of selected phytocompounds and reference compound using ProTox-II.

Parameters	3-Chloro-5-cholestene	Vitamin E	β-sitosterol	Squalene	6-Nitro-2-methylpyrrolo [2,3-g] quinoline	N- Acetyl cysteine
Toxicity class	4	5	4	5	3	5
LD50	1280 mg/kg	5000 mg/kg	890 mg/kg	5000 mg/kg	100 mg/kg	4400 mg/kg
Hepatotoxicity	Inactive (0.79)	Inactive (0.93)	Inactive (0.87)	Inactive (0.79)	Mild active (0.63)	Inactive (0.95)
Carcinogenicity	Mild active (0.50)	Inactive (0.79)	Inactive (0.60)	Inactive (0.76)	Active (0.78)	Inactive (0.71)
Cytotoxicity	Inactive (0.63)	Inactive (0.89)	Inactive (0.94)	Inactive (0.81)	Inactive (0.82)	Inactive (0.66)
Immunotoxicity	Active (0.99)	Inactive (0.95)	Active (0.91)	Inactive (0.99)	Inactive (0.73)	Inactive (0.99)
Mutagenicity	Inactive (0.87)	Inactive (0.95)	Inactive (0.98)	Inactive (0.98)	Active (0.98)	Active (0.76)
Neurotoxicity	Inactive (0.51)	Inactive (0.63)	Mild active (0.54)	Inactive (0.65)	Inactive (0.58)	Inactive (0.91)
Nrf2/ARE	Inactive (0.84)	Inactive (0.98)	Inactive (0.64)	Mild active (0.60)	Inactive (0.79)	Inactive (0.99)
MMP	Inactive (0.53)	Inactive (0.86)	Inactive (0.63)	Inactive (0.99)	Active (0.73)	Inactive (0.99)
p^53^	Inactive (0.88)	Inactive (0.97)	Inactive (0.99)	Inactive (1.0)	Inactive (0.71)	Inactive (0.99)
RYR	Inactive (0.92)	Inactive (0.98)	Inactive (0.98)	Inactive (0.96)	Inactive (0.96)	Inactive (0.98)
AChE	Inactive (0.77)	Inactive (0.93)	Inactive (0.87)	Inactive (0.81)	Inactive (0.75)	Inactive (0.95)
VGSC	Inactive (0.82)	Inactive (0.95)	Inactive (0.95)	Inactive (0.80)	Inactive (0.67)	Inactive (0.95)
GABA/NMDA	Inactive (0.55)	Inactive (0.92)	Inactive (0.96)	Inactive (0.81)	Inactive (0.61/0.94)	Inactive (0.96)
PXR	Mild active (0.50)	Inactive (0.92)	Inactive (0.92)	Mild active (0.51)	Inactive (0.73)	Inactive (0.92)
CYP2C9	Active (0.71)	Mild active (0.62)	Mild active (0.64)	Mild active (0.67)	Inactive (0.56)	Inactive (0.87)

No major hepatotoxicity, cytotoxicity, or carcinogenicity alerts were predicted for vitamin E, β-sitosterol, or squalene. In contrast, 6-nitro-2-methylpyrrolo [2,3-g] quinoline showed higher predicted toxicity, including mutagenic and carcinogenic activity, with a predicted LD50 value of 100 mg/kg.

Most compounds were predicted to be inactive against major neurotoxicity-associated targets, including acetylcholinesterase and voltage-gated sodium channels. Mild immunotoxicity and CYP2C9 interaction potential were observed for certain sterol-derived compounds.

#### Protein-protein interaction network analysis

A protein-protein interaction (PPI) network was generated using the STRING database to examine the association among venom-related targets (Figure 12). The network showed strong interactions among inflammatory mediators, coagulation-associated proteins, extracellular matrix remodelling enzymes, and signalling molecules, including TNF, IL6, AKT1, MAPK1, PTGS2, MMP2, MMP9, PLA2G2A, F2, F10, SERPINE1, and PLAT.

**FIGURE 12:**
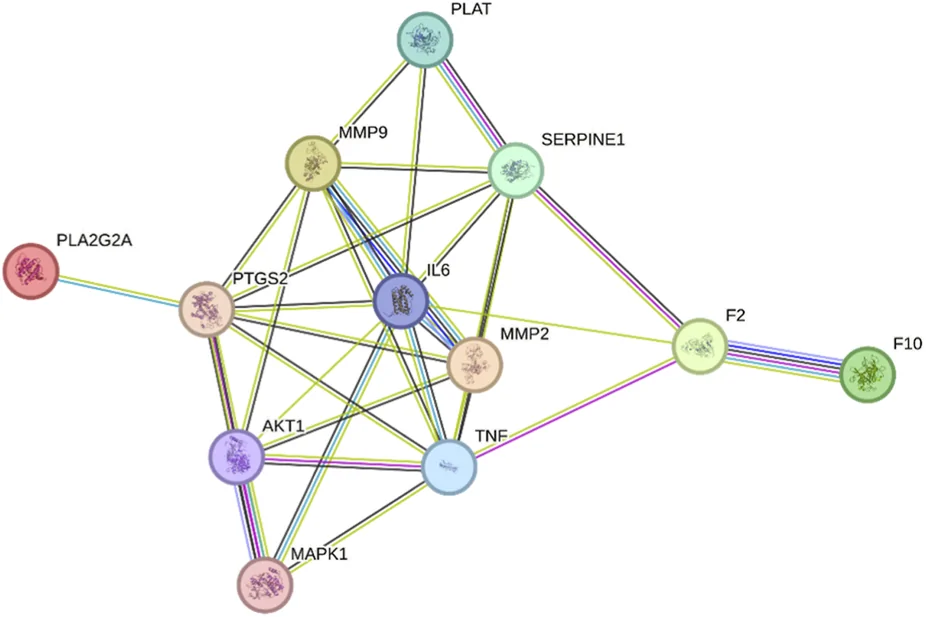
Protein-protein interaction network of venom-associated inflammatory, coagulation, and extracellular matrix-related targets generated using STRING database.

Inflammatory cytokines TNF and IL6 formed central nodes within the network, while MMP2 and MMP9 were closely associated with extracellular matrix degradation and hemorrhagic responses. Coagulation-related proteins such as F2, F10, SERPINE1, and PLAT also showed strong connectivity within the interaction map. Overall, the network indicated functional association among multiple pathways involved in venom-induced inflammation, coagulation dysfunction, and tissue injury.

#### KEGG pathway enrichment analysis

KEGG pathway enrichment analysis revealed significant enrichment of pathways associated with inflammation and coagulation (Figure 13). The most enriched pathways included complement and coagulation cascades, TNF signaling pathway, IL-17 signaling pathway, and cytokine-mediated inflammatory pathways.

**FIGURE 13:**
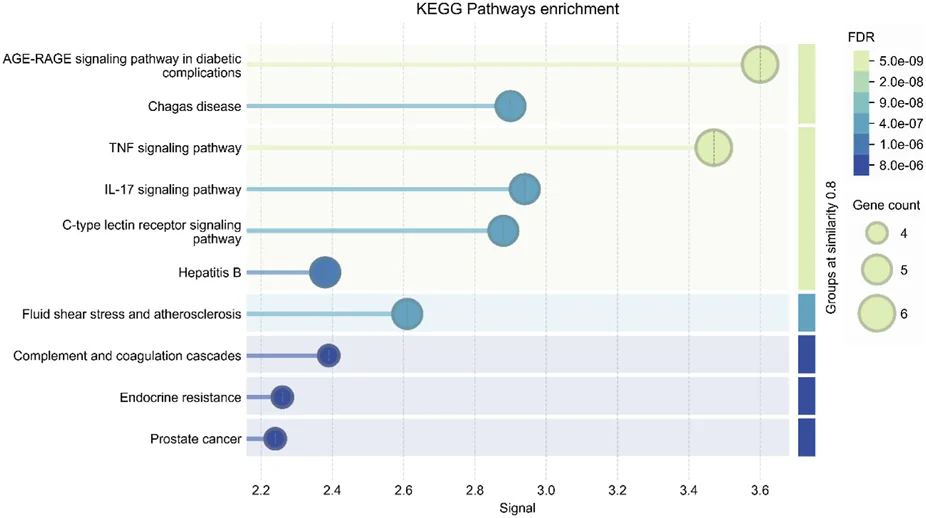
KEGG pathway enrichment analysis of venom-associated target genes showing significant enrichment of inflammatory and coagulation-related pathways.

Several target proteins, including F2, F10, SERPINE1, PLAT, TNF, IL6, PTGS2, AKT1, and MAPK1, were involved in these enriched pathways, indicating their association with venom-induced inflammatory and hemostatic disturbances.

#### Reactome pathway enrichment analysis

Reactome pathway analysis demonstrated significant enrichment of pathways related to cytokine signaling, hemostasis, platelet activation, extracellular matrix organization, and immune system regulation (Figure 14). Proteins including MMP2, MMP9, PLAT, SERPINE1, TNF, and IL6 were prominently represented within these pathways. The enrichment profile suggested coordinated involvement of inflammatory and coagulation-associated mechanisms during venom-induced pathology.

**FIGURE 14:**
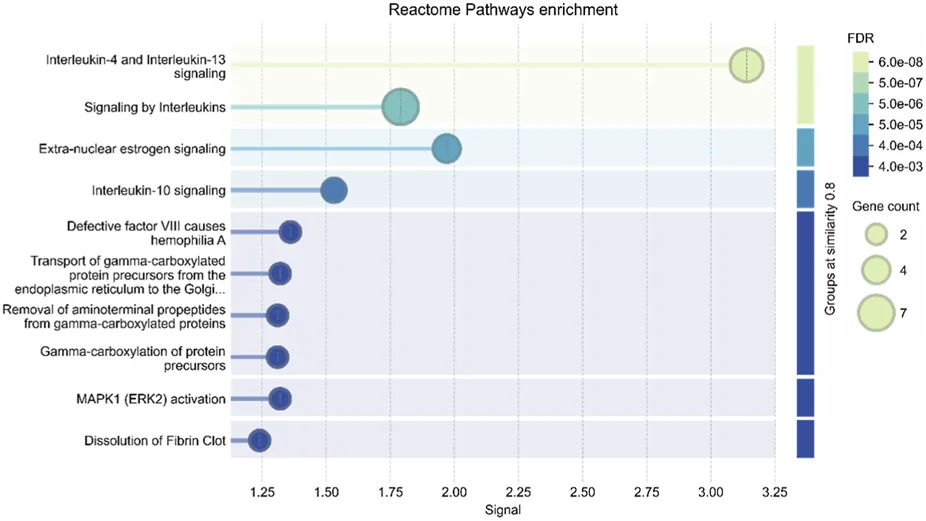
Reactome pathway enrichment analysis highlighting cytokine signalling, haemostasis, platelet activation, and extracellular matrix-associated pathways.

#### Gene Ontology biological process analysis

Gene Ontology biological process enrichment analysis identified significant enrichment of biological processes associated with inflammatory response, coagulation regulation, extracellular matrix disassembly, oxidative stress response, platelet activation, and cytokine-mediated signalling pathways (Figure 15).

**FIGURE 15:**
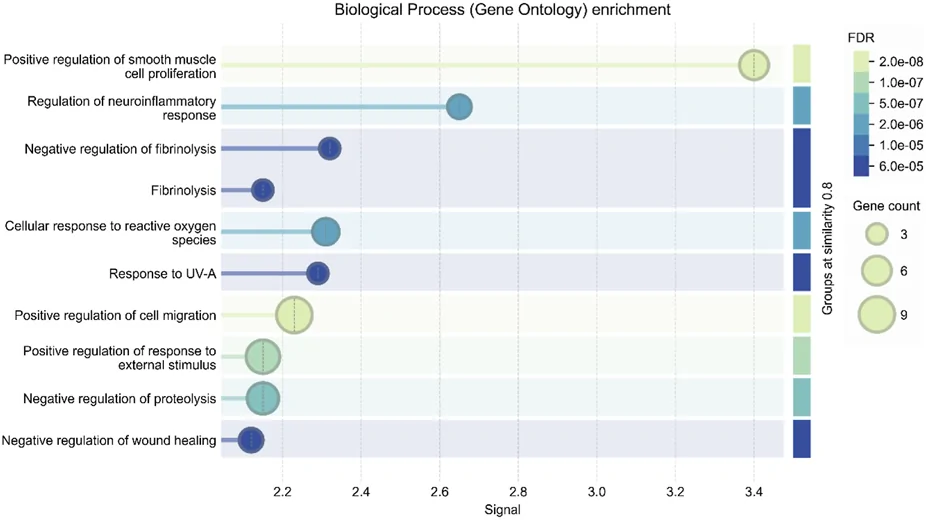
Gene Ontology biological process enrichment analysis of selected venom-associated targets related to inflammation, coagulation, oxidative stress, and extracellular matrix remodelling.

Key proteins including TNF, IL6, PTGS2, MAPK1, AKT1, MMP2, MMP9, F2, and F10 were involved in these enriched biological processes, highlighting their role in venom-associated inflammatory and vascular injury responses.

## Discussion

Snake envenomation remains a significant public health challenge in Asia-especially in rural areas, where traditional antivenom methods have limitations such as poor neutralization of localized effects, low protein coverage, high cost and adverse response (Gopalakrishnan et al., 2022; Gutiérrez et al., 2017; Williams et al., 2019). Based on this, the pursuit of safer and wide-spread Phyto therapeutics are now accelerating (Adrião et al., 2022). As new antivenom agents developed in isolation, we first investigated *Glycosmis pentaphylla* leaf extract-derived compounds in a comprehensive *in silico* approach.

Our docking analysis showed that several phytoconstituents from the leaf extract, particularly 3-chloro-5-cholestene and vitamin E, had positive binding affinity with all five major classes of viper venom proteins, superiorly or similarly to that of the N-acetyl cysteine (NAC) (Leão-Torres et al., 2021; Sunitha et al., 2011; Wang et al., 2018). The ligands had favourable docking coefficients and interacted with essential catalytic and substrate-binding functional residues and have potential to affect the action of enzymes to achieve neurotoxic, haemorrhagic, coagulopathy and systemic injury. Notably, the multi-target binding profile indicates that plant molecules represent a source of power to antagonize venom toxicity at several envenomation cascades (Albulescu et al., 2020; Gutierres et al., 2022; Tasoulis and Isbister, 2023). Similar multi-target docking strategies have previously identified phytochemicals capable of interacting with multiple toxin families including PLA_2_, SVSP and SVMP.

Eventhough, redocking-based validation is commonly employed to assess docking protocols and the present study focused on comparative virtual screening of phytochemicals followed by extensive molecular dynamics simulations of the highest-ranked complexes. Since molecular dynamics evaluates the structural stability of protein-ligand complexes under dynamic conditions over time, the reliability of the predicted binding modes was further supported through RMSD, RMSF, radius of gyration, solvent-accessible surface area, and hydrogen-bond analyses rather than relying solely on static docking predictions. A limitation of the present study is that docking protocol validation through native ligand redocking was not performed for all selected venom proteins because several structures lacked suitable co-crystallized small-molecule ligands for conventional RMSD-based validation. Therefore, the stability of the selected docked complexes was comprehensively evaluated using 100 ns molecular dynamics simulations.

Molecular dynamic analyses also established the stability and inhibition ability of the leading complexes. RMSD/Rg analyses showed that selective ligands (e.g., 3-chloro-5-cholestene) induced better conformational stability and densification of proteins such as PLA_2_ (1VIP) and SVMP (2E3X) than NAC. A reduction in activity of residues at functionally sensitive domains (such as catalytic dyads and zinc coordinate residue on RMSF profiles) illustrates the capability of ligands to reduce enzyme mobility in RMSF profiles. These observations were strengthened by hydrogen bonding and SASA results, which highlighted the persistence of interactions and deepening burial of specific ligands in the active clefts, so that solvent accessibility and the entry of the substrate are diminished. Taken together, their simulation data add mechanistic clues of how *G. pentaphylla* metabolites may inhibit the activity of venom protein. The stability of the selected protein-ligand complexes observed during the 100 ns simulations prompted further experimental validation using protease inhibition and membrane stabilization assays to evaluate whether the computational predictions could be translated into measurable biological activity (Alonso et al., 2023; Hollingsworth and Dror, 2018; Kato et al., 2021). Similar stabilization of catalytic residues and reduced conformational flexibility have been reported as indicators of stable inhibitor binding in protein-ligand MD simulations.

N-acetyl cysteine (NAC) was selected as the reference compound in the present study because of its well-established antioxidant, cytoprotective, and anti-inflammatory properties, which are relevant to several pathological events associated with *Daboia russelii* envenomation. NAC serves as a precursor of intracellular glutathione (GSH), thereby enhancing the cellular antioxidant defense system and reducing the excessive reactive oxygen species (ROS) generated following snake venom exposure. This antioxidant activity has been reported to attenuate venom-induced oxidative stress, lipid peroxidation, endothelial injury, and inflammatory tissue damage. In addition, the free sulfhydryl (-SH) group of NAC can interact with reactive oxygen species and has been reported to influence the activity of certain metal-dependent enzymes through redox regulation and metal-ion interactions, providing a mechanistic rationale for its use in studies involving snake venom metalloproteinases (SVMPs) (Aldini et al., 2018; Leão-Torres et al., 2021; Sunitha et al., 2011; Wang et al., 2018). Eventhough NAC is not a selective inhibitor of phospholipase A_2_ (PLA_2_), serine proteases (SVSPs), or other venom toxins, its ability to mitigate oxidative injury, preserve vascular integrity and also breaks the disulphide bonds of venom enzymes makes it a biologically relevant positive control for preliminary antivenom investigations. Moreover, NAC is a clinically approved, water-soluble molecule with well-characterized pharmacological and toxicological profiles, making it a reproducible and reliable reference compound for comparative evaluation (Sahasrabudhe et al., 2023; Sunitha et al., 2011). Varespladib is a well-established PLA_2_ inhibitor; however, because the present work investigated multiple venom toxin families rather than PLA_2_ alone, NAC was selected as a broad comparative reference owing to its reported antioxidant and cytoprotective properties. However, future studies should compare the identified compounds with toxin-specific inhibitors such as varespladib for PLA_2_ or established metalloproteinase inhibitors to further validate their therapeutic potential.

To provide preliminary biological validation of the computational findings, experiments were conducted using model systems relevant to venom-associated pathological mechanisms. Although these assays do not directly measure venom neutralization, they offer functional evidence for protease inhibition and membrane protection, two processes closely associated with snakebite pathology (Kalogeropoulos et al., 2019; Kini, 2005; Koh and Kini, 2025). The trypsin-casein assay was used to show concentration-dependent inhibition of proteolytic activity, with a maximum inhibition of 56.86% at 5 mg/mL. The observed inhibitory effects were statistically significant compared with the positive control, as determined by one-way ANOVA followed by Dunnett’s multiple comparison test, supporting a genuine biological response rather than experimental variation. As snake venom serine proteases play critical roles in coagulopathy and tissue degradation during envenomation, the suppression of trypsin activity observed supports experimental predictions made regarding phytoconstituents' interaction with protease targets. Results were also evident in the skim milk agar assay, in which the extract diminished the caseinolytic zone produced by trypsin.

Similar protease inhibitory activities have been reported for plant-derived flavonoids, terpenoids, and sterols from medicinal plants traditionally used against snakebite (Bala et al., 2023; Kumar et al., 2025). The present findings extend these observations by identifying β-sitosterol, vitamin E, and 3-chloro-5-cholestene as potential inhibitors of multiple toxin classes and by providing both computational and preliminary biological evidence supporting their activity.

In addition, the extract exhibited significant membrane stabilizing activity in an osmotic stress-induced erythrocyte hemolysis model (Ayinke et al., 2015; Yesmin et al., 2020). Although venom was not directly employed in this assay, stabilization of erythrocyte membranes is considered a relevant indicator of protection against membrane-disruptive toxins, particularly phospholipase A_2_ enzymes that contribute to hemolysis and inflammatory tissue damage following snakebite. The concentration-dependent reduction in hemolysis and the maximum stabilization of 39.23% observed at 5 mg/mL suggest that the extract may possess membrane-protective properties capable of mitigating toxin-induced cellular injury.

Eventhough the present study employed trypsin as a surrogate protease model and erythrocyte membrane stabilization assays to experimentally support the computational findings, these assays cannot fully reproduce the complexity of *D. russelii* envenomation. However, trypsin shares catalytic similarities with snake venom serine proteases, it cannot fully reproduce the biochemical complexity of *D. russelii* venom and making it a useful preliminary screening model. Moreover, membrane stabilization reflects one aspect of venom-induced cellular damage. Therefore, the present findings should be regarded as preliminary evidence supporting the inhibitory potential of *G. pentaphylla* constituents, which warrants further validation using purified venom enzymes, whole venom neutralization assays, and *in vivo* models.

Taken together, these experimental findings provide preliminary *in vitro* support for the computational predictions and suggest that *G. pentaphylla* extracts possess biologically relevant activities associated with venom-related pathological processes. To evaluate their therapeutic properties, the highest performing phytocompounds were screened on pharmacokinetic level and toxicity prediction on SwissADME and ProTox-II tools (Banerjee et al., 2018; Daina et al., 2017; Veber et al., 2002). Despite the Lipinski violations for hydrophobic sterol-like construct, most of the compounds showed acceptable bioavailability and drug-likeness. Most notably, both β-sitosterol and vitamin E exhibited relatively safe toxicity profiles without significant hepatotoxicity, cytotoxicity, or carcinogenicity advisories. Although 6-nitro-2-methylpyrrolo [2,3-g] quinoline showed high interaction potential with the specific venom proteins, its mutagenic and carcinogenic attributes may limit its therapeutic potential. The pharmacokinetic and toxic responses have favored β-sitosterol and vitamin E as promising lead agents to guide antivenom studies.

STRING-based protein-protein interaction and pathway enrichment analyses were conducted to investigate the broader biological significance of the detected venom-associated targets (Szklarczyk et al., 2023; Szklarczyk et al., 2025). The interaction network revealed powerful interactions between inflammatory cytokines, coagulation-related protein components, extracellular matrix remodelling enzymes, and intracellular signalling mediators, including TNF, IL6, AKT1, MAPK1, PTGS2, MMP2, MMP9, F2, and F10. These interconnected pathways closely mirror the main pathological sequelae of viper envenomation, including inflammation, hemorrhage, oxidative stress, endothelial injury, and coagulation dysfunction.

The PPI analysis was not intended to model direct interactions between phytocompounds and host proteins. Rather, it was performed to identify host signalling pathways known to be affected following inhibition of venom toxins. Therefore, the enrichment analysis complements the docking results by illustrating the downstream biological processes associated with venom pathology.

KEGG and Reactome enrichment analyses further highlighted the involvement of complement and coagulation cascades, TNF signalling, IL-17 signalling, cytokine-mediated inflammatory pathways, platelet activation, extracellular matrix degradation, and hemostasis-associated pathways (Kanehisa et al., 2025). Viper venoms are known to trigger thromboinflammatory processes as well, as indicated by the activation of inflammatory mediators and coagulation pathways, so these findings are relevant for biological understanding. Gene Ontology biological process analysis, similarly, revealed enrichment of systems related to inflammatory response, extracellular matrix disassembly, oxidative stress, cytokine signalling, and regulation of coagulation (Zhou et al., 2017). These pathway analyses collectively further substantiate the potential multi-target inhibitory activity of the selected phytocompounds on venom-induced pathological processes.

For the present investigation, all the major compounds of the three *G. pentaphylla* extracts were screened against several venom proteins with great methodicalness. Although docking affinity and simulation stability have been the main determinants for selection of the most promising ligands, additional phytoconstituents were found with strong inhibition potential (Kato et al., 2021). We focused on the molecular dynamics simulations of three main toxin classes: PLA_2_, SVMPs, and SVSPs(Alonso et al., 2023), responsible for venom toxicity and tissue damage. The sustained stability, compactness, and active-site interactions were shown during simulation, so it is possible that *G. pentaphylla* metabolites may possess broad-spectrum inhibitory potential against multiple venom protein families.

The results correlate with previous findings of antivenom effects reported from other plant phytochemicals such as flavonoids, terpenoids, sterols, and alkaloids derived from medicinal plants. Nonetheless, this study is among the first studies to thoroughly investigate *G. pentaphylla* phytoconstituents against multiple venom protein classes using a unified workflow (docking, molecular dynamics simulation, pharmacokinetic evaluation, toxicity prediction, and pathway enrichment analysis). The stabilization of the catalytic regions and the reduced conformational flexibility observed from several toxin classes indicate that these ligands can impact enzymatic catalysis by restricting substrate accessibility and changing the dynamics of the active site (Pagadala et al., 2017).

Limitations of the present study should be acknowledged. The biological validation was performed using trypsin-based model protease systems and erythrocyte membrane stabilization assays rather than purified *D. russelii* venom proteins or whole venom preparations. Consequently, the observed inhibitory effects should be considered preliminary evidence supporting the computational predictions rather than definitive proof of venom neutralization. Furthermore, the experimental studies were conducted using crude extracts, preventing direct attribution of activity to individual phytocompounds identified through GC-MS analysis. Future investigations should include venom-specific enzyme inhibition assays, direct venom neutralization studies, isolation of active constituents, and *in vivo* validation to establish therapeutic efficacy and safety.

Collectively, the integration of molecular docking, molecular dynamics simulation, pharmacokinetic evaluation, toxicity prediction, pathway enrichment analysis, and preliminary *in vitro* validation provide converging evidence supporting the therapeutic potential of *G. pentaphylla*-derived phytocompounds against venom-associated targets. The observed multi-target interactions, favorable safety predictions, protease inhibitory activity, and membrane-protective effects suggest that these phytoconstituents may serve as promising lead molecules for the development of adjunct therapies for snakebite envenomation.

## Conclusion

This study provides the first comprehensive *in silico* evidence for the therapeutic potential of *G. pentaphylla* constituents as promising lead compounds for future antivenom development against major venom proteins of *D. russelii*. Molecular docking and molecular dynamics analyses showed stable and favorable interactions between the key compounds, namely β-sitosterol, 3-chloro-5-cholestene, and vitamin E, with several venom-related targets including phospholipase A_2_, snake venom metalloproteinases, and serine proteases. The stability of these complexes observed, together with reduced conformational fluctuations and persistent active-site interactions throughout simulation, indicated that they may play a role in suppressing venom-associated enzymatic activity. Moreover, the pharmacokinetic and toxicity predictions showed that the acceptable drug-likeness characteristics and relatively safe toxicity profiles of several of the identified compounds also support their therapeutic viability. STRING interaction and pathway enrichment analyses indicated that the selected targets were closely associated with inflammatory, coagulation, oxidative stress, and extracellular matrix-associated pathways associated with snake envenomation pathology. Preliminary biological validation further supported the computational predictions. *Glycosmis pentaphylla* extract demonstrated significant inhibition of trypsin-mediated proteolysis in both liquid-phase and agar-based assays and exhibited moderate but significant erythrocyte membrane stabilization activity. These findings provide experimental evidence that the extract contains bioactive constituents capable of interfering with protease-mediated substrate degradation and protecting cellular membranes from stress-induced damage, two processes closely associated with venom pathology. Overall, the present research showed that the metabolites in this assay may provide great prospect as potential lead molecules for antivenom production from *G. pentaphylla*. While more validation will be required through *in vitro* and *in vivo* studies in future, this work provides a strong computational basis for the design of plant-based adjunct therapies to enhance the management of snakebite envenomation.

## Data Availability

The data presented in the study are deposited in the Figshare repository, DOI: 10.6084/m9.figshare.33771142.
