# Supplementary material for: Integrated computational investigation of Glycosmis pentaphylla-derived compounds against major venom proteins of Daboia russelii
Source: Front Mol Biosci. 2026 Sep 25;13:1906533. doi: 10.3389/fmolb.2026.1906533 (PMC13649486; doi:10.3389/fmolb.2026.1906533)
Supplement: Supplementary file 1 [file DataSheet1.pdf]

SUPPLEMENTARY FILE

Table 1. Docking scores of the different extracts of *Glycosmis pentaphylla* against nine snake venom proteins

Compounds	1DPY	1VIP	2PVT	2PYC	3S9B	3SBK	2E3X	3KVE	3UBU
1,4-Nonadiene, 2-Nitro-, (Z)-	-5.8	-5.6	-5.2	-5.4	-4.7	-4.7	-6.2	-6.1	-4.3
7-Oxabicyclo [4.1.0] Heptane, 3-Oxiranyl-	-5.7	-5.1	-4.1	-5.3	-4.6	-4.8	-4.8	-6	-4.2
Vitamin E	-8.0	-7.6	-7.4	-8.4	-7.5	-5	-6.6	-8.6	-5.7
3-Chloro-5-Cholestene	-8.9	-7.5	-6.5	-6.5	-7.2	-6.5	-6.3	-8.6	-5.9
Cis-1,3-Cyclohexanedicarbonitrile	-5.6	-5.1	-4.6	-5.2	-4.7	-5.7	-6.3	-5.8	-4.4
10-Dodecyn-1-ol	-5.0	-5	-4.2	-4.5	-4	-4.5	-4.3	-4.9	-3.8
Phytol	-6.2	-5.2	-5.8	-5.7	-5.6	-4.9	-4.4	-6.7	-4.2
2-Methyl-6-Methylene-Octa-1,7-Dien-3-ol	-5.3	-5.1	-5	-5.4	-4.7	-4.6	-5.2	-5.6	-4.6
3,7,11,15-Tetramethyl-2-Hexadecen-1-ol	-6.3	-5.5	-5.9	-5.8	-5.5	-5.1	-5.5	-6.6	-4.2
D-Mannitol, 1-Decylsulfonyl-	-6.3	-5.3	-5.5	-5.5	-5.6	-6.2	-5.8	-5.9	-5.6
3-Nonen-1-ol, (Z)-	-5.5	-4.9	-47	-4.8	-4.3	-4.5	-5	-5	-3.7
9-Octadecynoic Acid	-5.9	-5	-5.4	-5.6	-4.7	-4.5	-4	-6.1	-4.2
1-Tetradecyne	-5.3	-4.3	-3.5	-5.1	-3.6	-4.7	-3.7	-5.5	-3.1
Decanal	-5.1	-4.7	-4.1	-4.5	-4.2	-4.5	-4.6	-5	-3.4
N-Hexadecanoic Acid	-5.4	-5.1	-4.9	-5.0	-4.4	-4.9	-4.7	-5.8	-3.6
Cyclohexane	-4.2	-3.6	-3.6	-4	-3.8	-8.4	-4.6	-4.4	-3.3
Ether, 1-Butyl Vinyl Methyl	-4.1	-3.8	-3.9	-4.1	-3.9	-3.9	-4.1	-4.4	-3.4
Pentadecanoic Acid	-5.7	-5	-5	-3.8	-4.6	-5.1	-5	-5.8	-4
(Z)6, (Z)9-Pentadecadien-1-ol	-5.7	-5	-4.9	-5.3	-4.9	-5	-4.9	-5.9	-4.4

1,2:4,5:9,10-Triepoxydecane	-4.9	-4.9	-4.4	-4.4	-4	-4.4	-4.8	-5.1	-4.3
2-Chloro-6-methyl-3-(2-nitroethenyl) quinoline	-7.4	-7.1	-6.7	-6.2	-6.2	-6.5	-6.8	-8.6	-5.8
6-Nitro-2-Methylpyrrolo[2,3-G] Quinoline	-7.3	-8	-6.8	-7.4	-6.6	-6.7	-7.7	-8.9	-6
Squalene	-7.9	-6.5	-6.5	-7.5	-4.9	-5	-5.2	-8.1	-4.6
Heptacosane	-5.3	-5.4	-4.9	-3.9	-5.1	-4.6	-5.7	-5.6	-4.4
2-Bromotetradecane	-5.2	-5	-4.6	-4.4	-4.1	-4.7	-4.7	-5	-3.6
Octacosane	-5.6	-4.9	-5.1	-5.2	-3.9	-4.8	-3.4	-5.6	-3.6
Beta- sitosterol	-9.3	-8.2	-7.4	-6.8	-7.6	-7	-8.1	-9.3	-6.3
Cyclopropene, 3,3-Diethyl-	-4.5	-4.1	-3.8	-4.1	-4.2	-4	-4.3	-4.4	-3.5
3-Methoxy-1-Pentene	-4	-3.6	-3.5	-3.7	-3.8	-3.7	-3.9	-4.2	-3.8
Oxalic Acid	-5.9	-5.2	-5.2	-5.3	-5.1	-6.1	-5.7	-6.3	-4.7
cyclopropane, (2-Methylenebutyl)-	-5.1	-4.7	-4.4	-4.6	-4.1	-4.5	-4.6	-4.8	-3.7
1-Nitrododecane,	-5.5	-4.8	-4.4	-3.9	-4.6	-5	-4.6	-5.3	-3.6

Table 2. Best docking scores (kcal/mol) and interacting residues of *Glycosmis pentaphylla* extracts (MME, MPE, MHE) against Phospholipase A_2 (PLA_2) proteins.

Sl. No.	Extract	Compound ID	PDB ID	Binding Energy	Key Interactions
1.	MME	3-chloro-5- cholestene-17750989	1DPY	-8.9	Trp A: 19, Val A: 23, Leu A: 2, Tyr A: 52
2.	MME	Vitamin E - 14985	1DPY	-8	Trp A: 19, His A: 48, Phe A: 5, Val A: 23, Leu A: 2
3.	MME	Phytol - 5280435	1DPY	-6.2	Val A: 23, Phe A: 5, Trp A: 19; Leu A: 2, Gly A: 30
4.	MME	D-Mannitol, 1-Decylsulfonyl-568528	1DPY	-6.3	Cys A: 29, Gly A: 30, His A: 48, Phe A: 101, Phe A: 5, Asp A: 49, Trp A: 19, Lys A: 6, Leu A: 2
5.	MPE	6-Nitro-2-Methylpyrrolo[2,3-G] Quinoline –614864	1DPY	-7.3	Asp A: 4, His A: 50

6.	MPE	Squalene - 638072	1DPY	-7.9	Ile A: 9, Phe A: 5, Cys A: 45, Cys A: 29, Tyr A: 52, His A: 48, Leu A: 2
7.	MPE	Beta-Sitosterol–222284	1DPY	-9.3	Phe A: 101, Phe A: 5, Leu A: 2, His A: 48, Tyr A: 52
8.	MHE	Oxalic acid- 6420622	1DPY	-5.9	Val A: 23, Phe A: 5, Lys A: 6, Trp A: 19, Leu A: 2, His A: 48, Gly A: 30
9.	MHE	1-Nitrododecane-85629	1DPY	-5.5	Gly A: 30, His A: 48, Phe A: 5, Lys A: 6, Leu A: 2, Trp A: 19
10.	MME	3-chloro-5- cholestene-17750989	1VIP	-7.5	Lys A:69, Phe A:5, Ala A:6, Pro A:18, Ile A:9
11.	MME	Vitamin E - 14985	1VIP	-7.6	Pro A:18, Cys A:29, Leu A:19, CysA:45, PheA:3
12.	MME	Phytol - 5280435	1VIP	-5.2	Leu A:19, Leu A:2, Tyr A:28, Phe A:5, Gly A:30
13.	MME	D-Mannitol, 1-Decylsulfonyl-568528	1VIP	-5.3	Pro A:18, Ile A:9, Leu A:2, Phe A:5, Ala A:6, His A:48, Asp A:49
14.	MPE	6-Nitro-2-Methylpyrrolo[2,3-G] Quinoline –614864	1VIP	-8	Gly A: 30, His A: 48, Leu A: 2, Ala A: 6, Pro A: 18, Cys A: 45
15.	MPE	Squalene - 638072	1VIP	-6.5	Phe A: 5, Cys A: 45, His A: 48, Ala A: 6, Pro A: 18, Trp A: 31
16.	MPE	Beta-Sitosterol–222284	1VIP	-8.2	His A: 48, Asp A: 49, Cys A: 45, Phe A: 5, Pro A: 18, Ala A: 6, Phe A: 3
17.	MHE	Oxalic acid- 6420622	1VIP	-5.2	Phe A: 3, Phe A: 5, Leu A: 2, Ala A: 6, Cys A: 45, His A: 48, Pro A: 18, Ser A: 23
18.	MHE	1-Nitrododecane-85629	1VIP	-4.8	Gly A: 30, His A: 48, Phe A: 5, Ala A: 6, Leu A: 2, Pro A: 18
19.	MME	3-chloro-5- cholestene-17750989	2PVT	-6.5	Lys A: 69, Pro A: 68, Tyr A: 52

20.	MME	Vitamin E - 14985	2PVT	-7.4	Asp A: 49, Leu A: 2, Ala A: 18, Ile A: 19, Phe A: 5
21.	MME	Phytol - 5280435	2PVT	-5.8	Phe A: 5, Leu A: 2, Ile A: 19, Ala A: 18
22.	MME	D-Mannitol, 1-Decylsulfonyl-568528	2PVT	-5.5	His A: 48, Phe A: 5, Ala A:18, Leu A: 2, Cys A: 29, Tyr A: 22, Lys A: 69, Cys A: 45, Asp A: 49, Gly A: 30
23.	MPE	6-Nitro-2-Methylpyrrolo[2,3-G] Quinoline –614864	2PVT	-6.8	Asp A: 49, Tyr A: 52, His A: 48, Phe A: 5, Cys A: 45
24.	MPE	Squalene - 638072	2PVT	-6.5	Phe A: 5, Ala A: 18, Leu A: 2, Tyr A: 52, Pro A: 68, Cys A: 132, Phe A: 46
25.	MPE	Beta-Sitosterol–222284	2PVT	-7.4	Leu A: 2, Ala A: 18, Ile A: 19, Gly A: 30
26.	MHE	Oxalic acid- 6420622	2PVT	-5.2	His A: 48, Gly A: 30, Tyr A: 52, Phe A: 5, Cys A: 132, Ala A: 18, Tyr A: 28, Cys A: 30, Phe A: 46
27.	MHE	1-Nitrododecane-85629	2PVT	-4.4	Ala A: 18, Leu A: 2, Phe A: 5, Ile A: 19
28.	MME	3-chloro-5- cholestene-17750989	2PYC	-6.5	Lys A: 69, Tyr A: 52, Tyr A: 28, Phe A: 46, Pro A: 68
29.	MME	Vitamin E - 14985	2PYC	-8.4	Ile A: 9, Cys A: 29, Cys A: 45, Ala A: 18, Leu A: 2, Phe A: 5, His A: 48, Lys A: 69, Tyr A: 52, Pro A: 68
30.	MME	Phytol - 5280435	2PYC	-5.7	Pro A: 68, Tyr A: 52, Leu A: 2
31.	MME	D-Mannitol, 1-Decylsulfonyl-568528	2PYC	-5.5	Asp A: 49, His A: 48, Lys A: 69, Gly A: 30, Cys A: 45, Ile A: 9, Leu A: 2, Phe A: 5, Ala A: 18, Ile A: 19
32.	MPE	6-Nitro-2-Methylpyrrolo[2,3-G] Quinoline –614864	2PYC	-7.4	Asp A: 49, His A: 48, Tyr A: 52, Phe A: 5, Cys A: 45
33.	MPE	Squalene - 638072	2PYC	-7.5	Ile A: 19, Ala A: 18, Leu A: 17, Phe A: 5, His A: 48, Lys A: 69, Tyr A: 52
34.	MPE	Beta-Sitosterol – 222284	2PYC	-6.8	Lys A: 69, Pro A: 68, Cys A: 133, Leu A: 130

35.	MHE	Oxalic acid- 6420622	2PYC	-5.3	Ala A: 18, Phe A: 5, His A: 48, Asp A: 49, Gly A: 30, Tyr A: 52
36.	MHE	1-Nitrododecane-85629	2PYC	-3.9	Leu A: 2, Ile A: 9, Ala A: 18, Phe A: 5, Ile A: 19, Cys A: 45, Cys A: 29, His A: 48, Lys A: 69

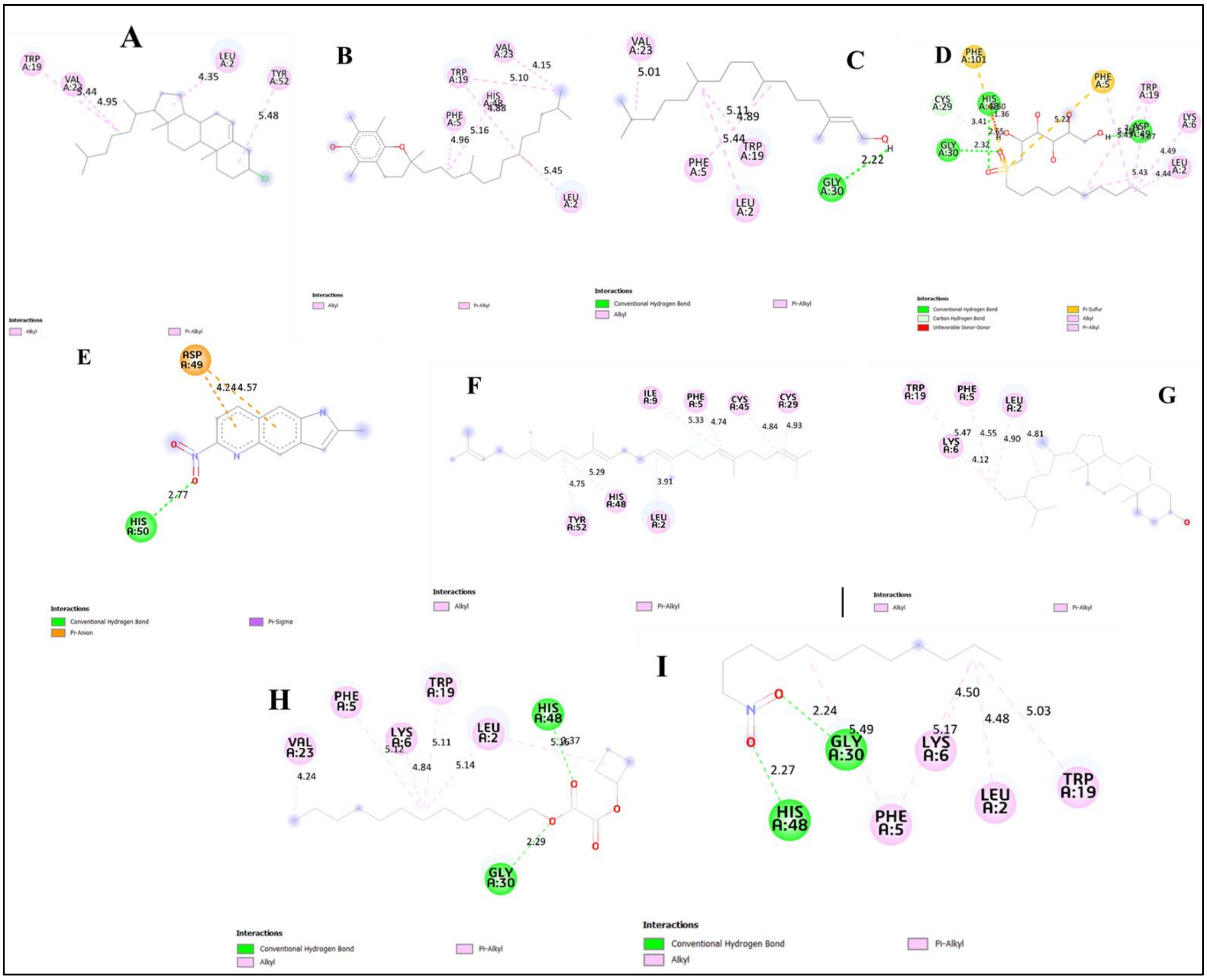

Figure 2(A-I). 2D interaction diagrams of selected ligands from different extracts (A-3-chloro-5- cholestene, B- Vitamin E, C- Phytol, D- D-Mannitol, 1-Decylsulfonyl, E-6-Nitro-2-Methylpyrrolo[2,3-G] Quinoline, F- Squalene, G- Beta-Sitosterol, H- Oxalic acid, I- 1-Nitrododecane) with venom-related protein 1DPY showing hydrogen bonding, hydrophobic, and other key interactions.

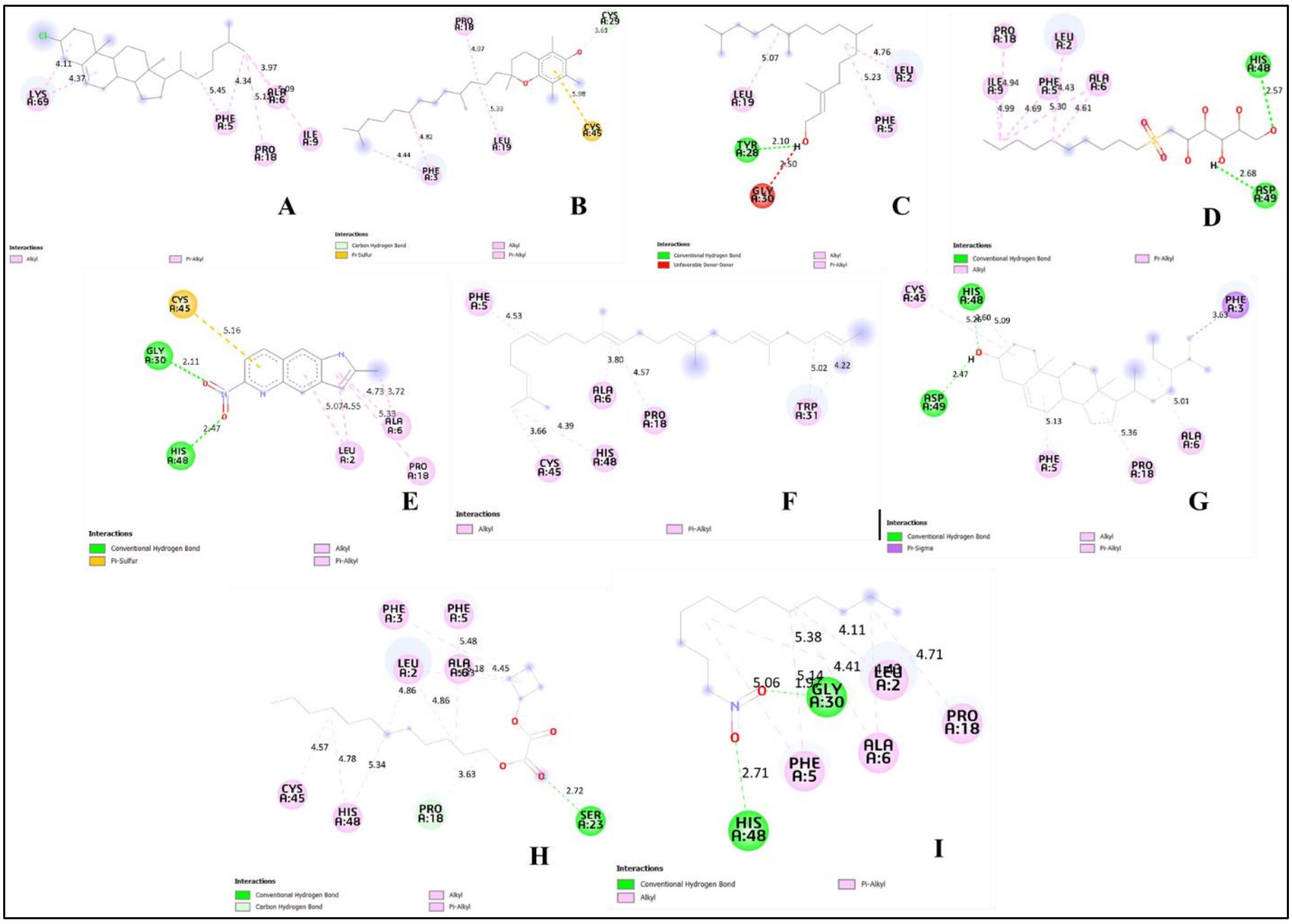

Figure 3(A-I). 2D interaction diagrams of selected ligands from different extracts (A-3-chloro-5- cholestene, B- Vitamin E, C- Phytol, D- D-Mannitol, 1-Decylsulfonyl, E-6-Nitro-2-Methylpyrrolo[2,3-G] Quinoline, F- Squalene, G- Beta-Sitosterol, H- Oxalic acid, I- 1-Nitrododecane) with venom-related protein 1VIP showing hydrogen bonding, hydrophobic, and other key interactions.

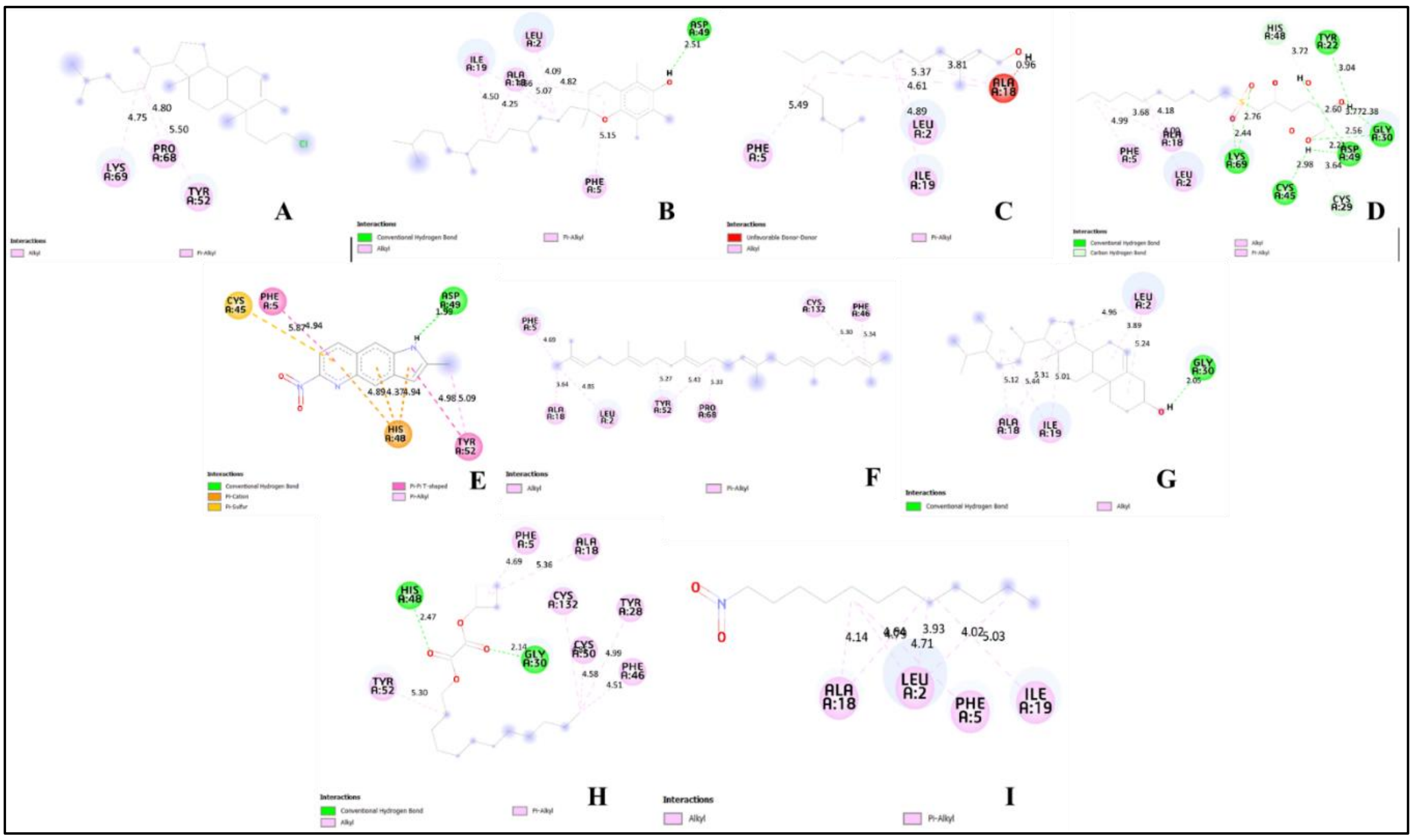

Figure 4(A-I). 2D interaction diagrams of selected ligands from different extracts (A-3-chloro-5- cholestene, B- Vitamin E, C- Phytol, D- D-Mannitol, 1-Decylsulfonyl, E-6-Nitro-2-Methylpyrrolo[2,3-G] Quinoline, F- Squalene, G- Beta-Sitosterol, H- Oxalic acid, I- 1-Nitrododecane) with venom-related protein 2PVT showing hydrogen bonding, hydrophobic, and other key interactions.

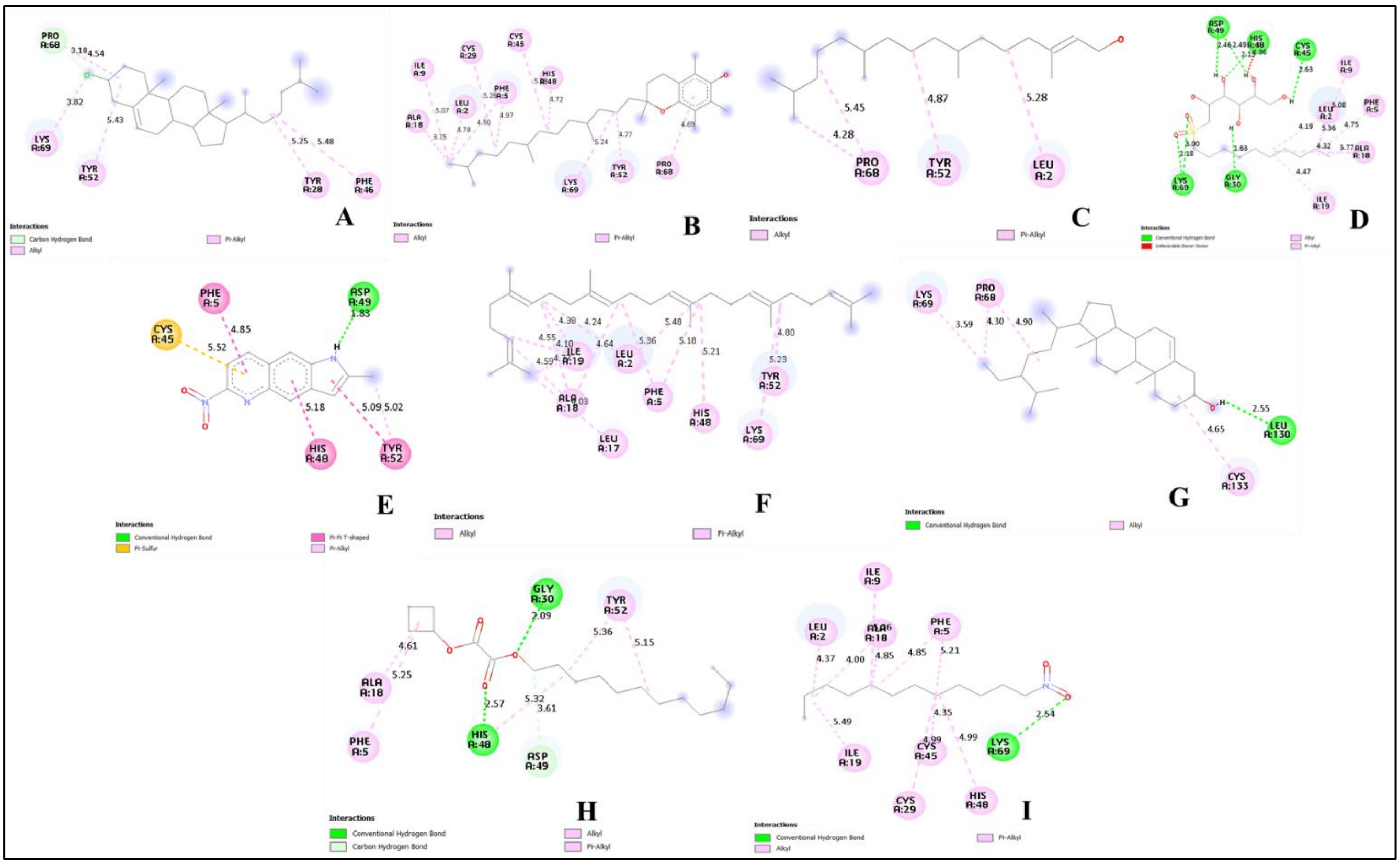

Figure 5(A-I). 2D interaction diagrams of selected ligands from different extracts (A-3-chloro-5- cholestene, B- Vitamin E, C- Phytol, D- D-Mannitol, 1-Decylsulfonyl, E-6-Nitro-2-Methylpyrrolo[2,3-G] Quinoline, F- Squalene, G- Beta-Sitosterol, H- Oxalic acid, I- 1-Nitrododecane) with venom-related protein 2PYC showing hydrogen bonding, hydrophobic, and other key interactions.

Table 3. Best docking scores (kcal/mol) and interacting residues of Glycosmis extracts against Snake Venom Metalloproteinase (SVMP) and Snake Venom Serine Protease (SVSP).

Sl. No.	Extract	Compound ID	Protein	PDB ID	Binding Energy	Key Interactions
1.	MME	3-chloro-5-cholestene-17750989	SVSP	3S9B	-7.2	Arg A: 62, Leu A: 92
2.	MME	Vitamin E - 14985	SVSP	3S9B	-7.5	Tyr A: 34, Lys A: 73, Lys A: 67
3.	MME	Phytol - 5280435	SVSP	3S9B	-5.6	Ala A: 56, Cys A: 91, Phe A: 90, Leu A: 92, Trp A: 237, Tyr A: 89, Arg A: 245A, Ile A: 241, Val A: 245C

4.	MME	D-Mannitol, 1-Decylsulfonyl-568528	SVSP	3S9B	-5.6	Ile A: 200, Phe A: 29, Val A: 121, Arg A: 135, Gly A: 207, Asn A: 202
5.	MPE	6-Nitro-2-Methylpyrrolo[2,3-G] Quinoline – 614864	SVSP	3S9B	-6.6	Asp A: 59, Asn A: 97, Ala A: 56, Phe A: 90
6.	MPE	Squalene - 638072	SVSP	3S9B	-4.9	Leu A: 186, Ile A: 185, Phe A: 161, Val A: 132, Val A: 163, Lys A: 164
7.	MPE	Beta-Sitosterol–222284	SVSP	3S9B	-7.6	Phe A: 95A, Leu A: 99, Trp A: 173, Gly A: 216
8.	MHE	Oxalic acid-6420622	SVSP	3S9B	-5.1	Trp A: 173, Trp A: 172, Val A: 227, His A: 57, Gly A: 215, Leu A: 99, Ala A: 214, Ser A: 195
9.	MHE	1-Nitrododecane-85629	SVSP	3S9B	-4.6	Asn A: 97, Leu A: 99, Ala A: 214, Trp A: 173, Tyr A: 172, Val A: 227
10.	MME	3-chloro-5-cholestene-17750989	SVSP	3SBK	-6,5	Leu A: 92
11.	MME	Vitamin E - 14985	SVSP	3SBK	-5	Phe A: 161, Val A: 132, Leu A: 186
12.	MME	Phytol - 5280435	SVSP	3SBK	-4.9	Trp A: 173, Ala A: 214, Val A: 227, Tyr A: 172, Val A: 174, Leu A: 99, His A: 57
13.	MME	D-Mannitol, 1-Decylsulfonyl-568528	SVSP	3SBK	-6.2	Leu A: 99, Tyr A: 172, Ala A: 214, Trp A: 173, Val A: 174, Val A: 227, Gly A: 216, Cys A: 191
14.	MPE	6-Nitro-2-Methylpyrrolo[2,3-G] Quinoline – 614864	SVSP	3SBK	-6.7	Ala A: 56, Phe A: 90, Leu A: 92
15.	MPE	Squalene - 638072	SVSP	3SBK	-5	Leu A: 186, Lys A: 186A, Ile A: 185, Phe A: 161
16.	MPE	Beta-Sitosterol–222284	SVSP	3SBK	-7	Trp A: 173, Leu A: 99
17.	MHE	Oxalic acid-6420622	SVSP	3SBK	-6.1	Ala A: 214, Cys A: 220, Tyr A: 172, Val A: 227, Leu A: 99, His A: 57, Ser A: 195, Gly A: 193, His A: 192

18.	MHE	1-Nitrododecane-85629	SVSP	3SBK	-5	Tyr A: 172, Val A: 227, Ala A:214, Val A: 174, Leu A: 99, Gly A: 215, Ser A: 195, His A: 57
19.	MME	3-chloro-5-cholestene-17750989	SVMP	2E3X	-6.3	Pro A:316, Tyr A:196, Arg A:195
20.	MME	Vitamin E - 14985	SVMP	2E3X	-6.6	Arg B:10, Glu A:340, Pro A:382
21.	MME	Phytol - 5280435	SVMP	2E3X	-4.4	Asn A:69
22.	MME	D-Mannitol, 1-Decylsulfonyl-568528	SVMP	2E3X	-5.8	Leu A:296, Arg A:275, His A:95, Lys A:199, Cys A:120, Arg A:85, Asp A:96
23.	MPE	6-Nitro-2-Methylpyrrolo[2,3-G] Quinoline – 614864	SVMP	2E3X	-7.7	Gly A: 335, Ile A: 68, Trp A: 84
24.	MPE	Squalene - 638072	SVMP	2E3X	-5.2	Lys B: 75, Cys B: 119
25.	MPE	Beta-Sitosterol–222284	SVMP	2E3X	-8.1	Lys A: 380, Lys B: 53, Arg B: 10, Tyr B: 11
26.	MHE	Oxalic acid-6420622	SVMP	2E3X	-5.7	Cys A: 120, Pro A: 198, Arg A: 124, Pro A: 316, Ala A: 122, Pro A: 399
27.	MHE	1-Nitrododecane-85629	SVMP	2E3X	-4.6	Tyr B: 9, Arg B: 10, Phe B: 12, Cys A: 383, Ser B: 44

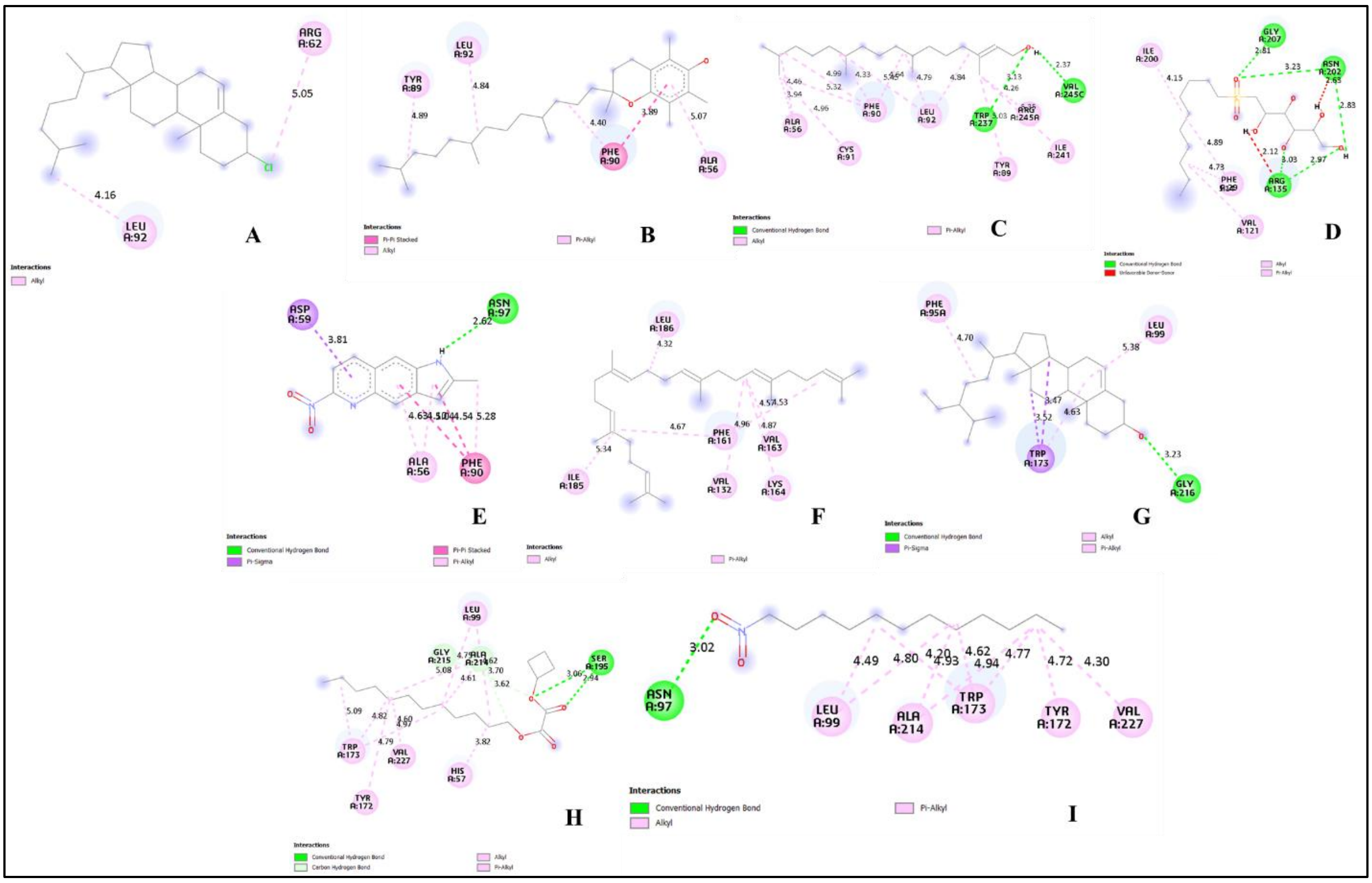

Figure 6 (A-I). 2D interaction diagrams of selected ligands from different extracts (A-3-chloro-5- cholestene, B- Vitamin E, C- Phytol, D- D-Mannitol, 1-Decylsulfonyl, E-6-Nitro-2-Methylpyrrolo[2,3-G] Quinoline, F- Squalene, G- Beta-Sitosterol, H- Oxalic acid, I- 1-Nitrododecane) with venom-related protein 3S9B showing hydrogen bonding, hydrophobic, and other key interactions.

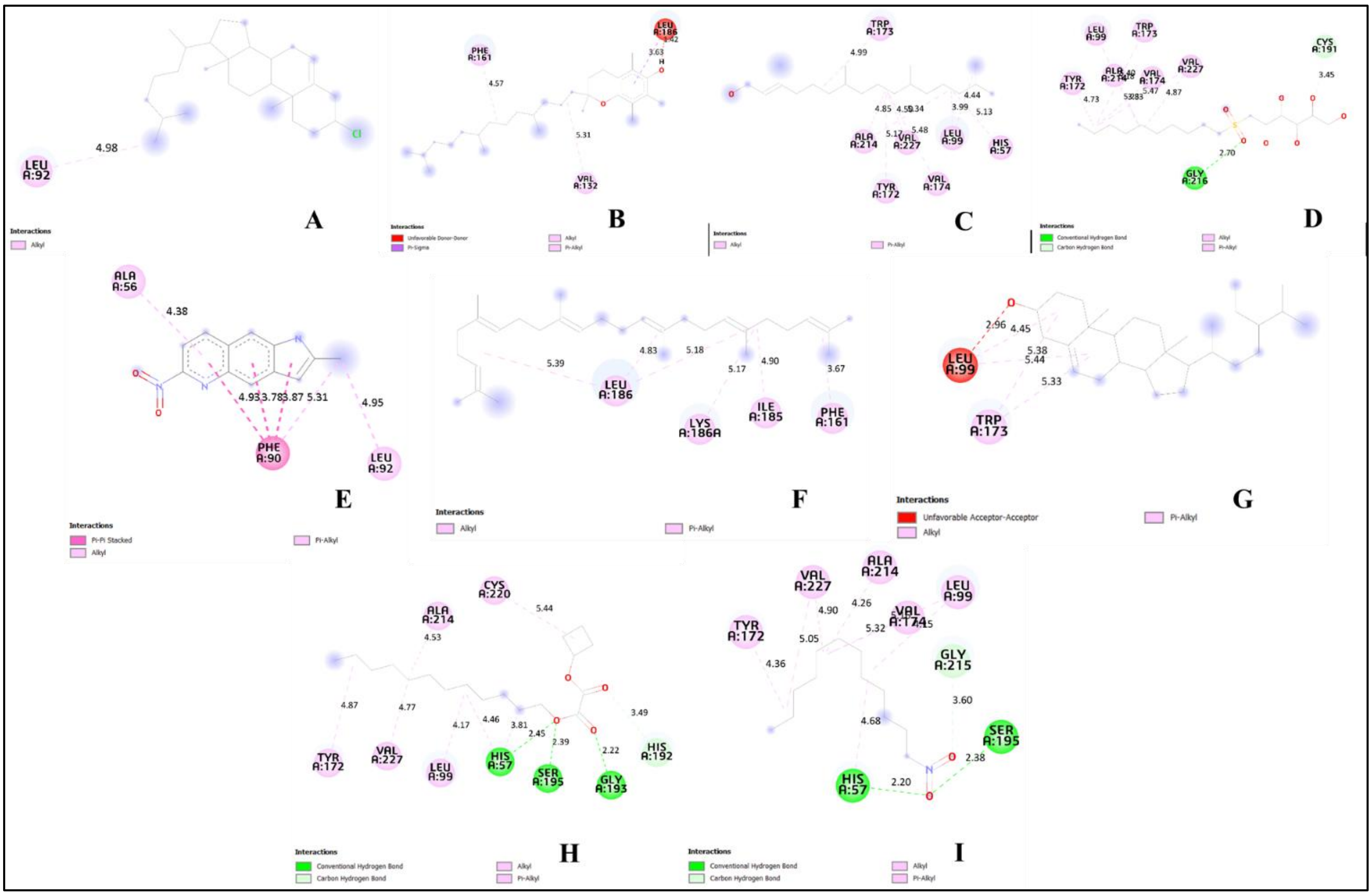

Figure 7 (A-I). 2D interaction diagrams of selected ligands from different extracts (A-3-chloro-5- cholestene, B- Vitamin E, C- Phytol, D- D-Mannitol, 1-Decylsulfonyl, E-6-Nitro-2-Methylpyrrolo[2,3-G] Quinoline, F- Squalene, G- Beta-Sitosterol, H- Oxalic acid, I- 1-Nitrododecane) with venom-related protein 3SBK showing hydrogen bonding, hydrophobic, and other key interactions.

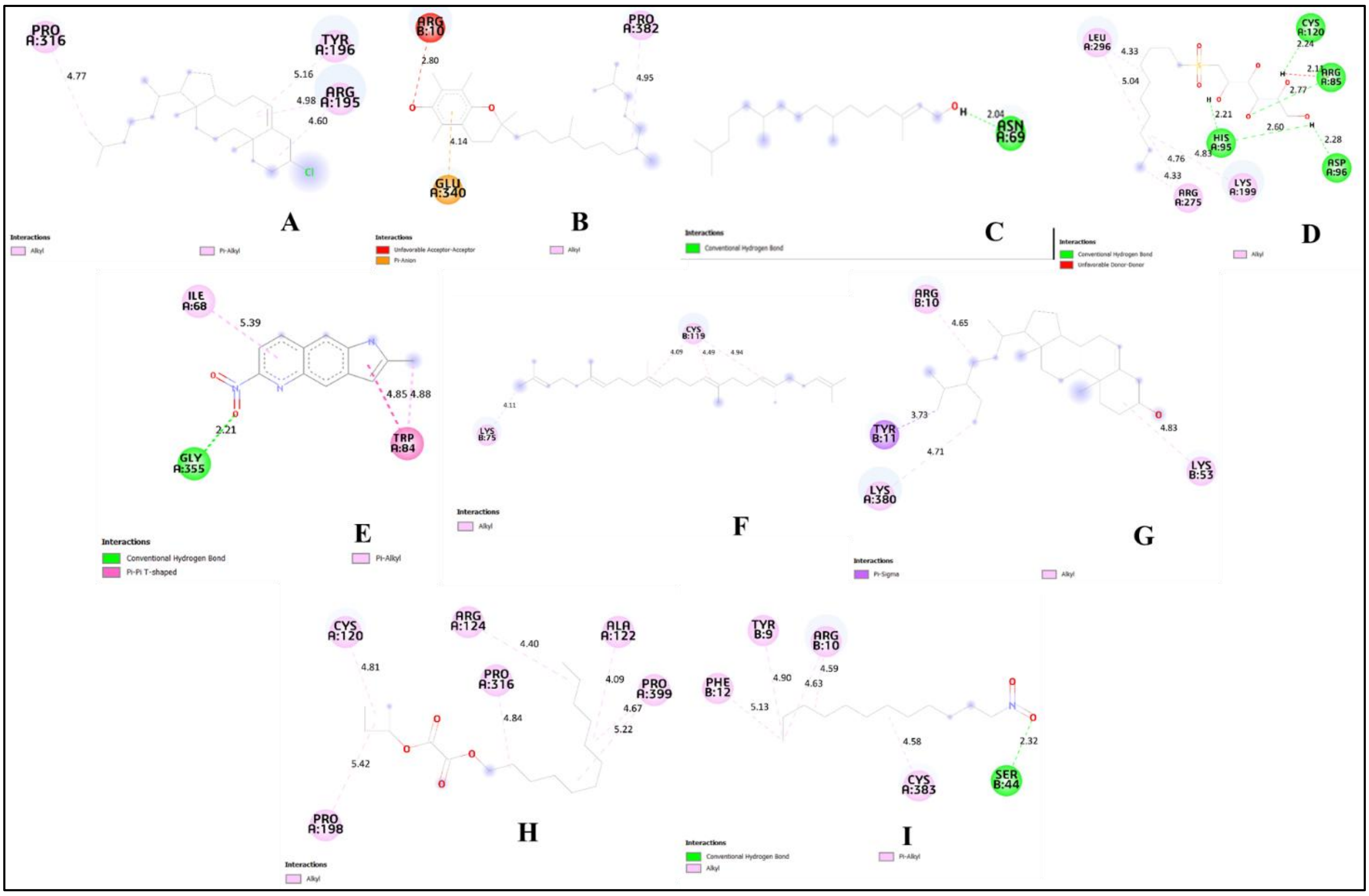

Figure 8 (A-I). 2D interaction diagrams of selected ligands from different extracts (A-3-chloro-5- cholestene, B- Vitamin E, C- Phytol, D- D-Mannitol, 1-Decylsulfonyl, E-6-Nitro-2-Methylpyrrolo[2,3-G] Quinoline, F- Squalene, G- Beta-Sitosterol, H- Oxalic acid, I- 1-Nitrododecane) with venom-related protein 2E3X showing hydrogen bonding, hydrophobic, and other key interactions.

Table 4. Best docking scores (kcal/mol) and interacting residues of Glycosmis extracts against Snake C-type Lectin-like Protein (SNACLEC) and L-amino Acid Oxidase (LAAO).

Sl. No.	Extract	Compound ID	Protein	PDB ID	Binding Energy	Key Interactions
1.	MME	3-chloro-5-cholestene-17750989	LAAO	3KVE	-8.6	Lys A: 387, Pro C: 414, Tyr C: 413, Pro A: 414, Tyr A: 413
2.	MME	Vitamin E - 14985	LAAO	3KVE	-8.6	Pro B: 414, Phe B: 394, Tyr B: 413, Pro D: 414, Tyr D: 413
3.	MME	Phytol - 5280435	LAAO	3KVE	-6.7	Tyr D: 413, Tyr B: 413, Phe B: 394, Gln D: 409, Pro B: 414
4.	MME	D-Mannitol, 1-Decylsulfonyl-568528	LAAO	3KVE	-5.9	Cys B: 412, Gln B: 409, Tyr B: 413 Pro D: 414, Glu D: 80, Tyr D: 413
5.	MPE	6-Nitro-2-Methylpyrrolo[2,3-G] Quinoline – 614864	LAAO	3KVE	-8.9	Met D: 89, Arg D: 90, Pro D: 88, Glu D: 457, Arg D: 71

6.	MPE	Squalene - 638072	LAAO	3KVE	-8.1	Pro D: 414, Tyr B: 413, Ala D: 390, Phe D:394<br>Pro B: 414, Tyr D: 413, Val B: 366
7.	MPE	Beta-Sitosterol–222284	LAAO	3KVE	-9.3	Tyr B: 413, Pro D: 414, Lys D: 387, Tyr D: 413, Pro B: 414
8.	MHE	Oxalic acid-6420622	LAAO	3KVE	-6.3	Phe B: 227, Trp B: 465, Ile B: 374, Ile B: 430, Met B: 43, Arg B: 71, Ile B: 466, Val B: 72
9.	MHE	1-Nitrododecane-85629	LAAO	3KVE	-5.3	Tyr B: 413, Pro B: 414, Pro D: 414, Tyr D: 413, Ser D: 332
10.	MME	3-chloro-5-cholestene-17750989	SNACLEC	3UBU	-5.9	Lys A: 75
11.	MME	Vitamin E - 14985	SNACLEC	3UBU	-5.7	Tyr A: 97, Lys A: 100, Leu A: 115
12.	MME	Phytol - 5280435	SNACLEC	3UBU	-4.2	Asn A: 116, Leu A: 115, Asp B: 97, Thr B: 95
13.	MME	D-Mannitol, 1-Decylsulfonyl-568528	SNACLEC	3UBU	-5.6	Lys B: 30, Trp B: 25, Ser A: 80, Ser B: 71, Glu A: 82, Thr A: 81
14.	MPE	6-Nitro-2-Methylpyrrolo[2,3-G] Quinoline – 614864	SNACLEC	3UBU	-6	Ser A: 80, Ser B: 71, Asp B: 76
15.	MPE	Squalene - 638072	SNACLEC	3UBU	-4.6	Lys A: 30, Lys A: 71<br>Trp A: 25
16.	MPE	Beta-Sitosterol–222284	SNACLEC	3UBU	-6.3	Leu A: 117, Tyr A: 97
17.	MHE	Oxalic acid-6420622	SNACLEC	3UBU	-4.7	Ser A: 80, Ser B: 71, Asn B: 72, Trp B: 25, Lys B: 30
18.	MHE	1-Nitrododecane-85629	SNACLEC	3UBU	-3.6	Lys A: 128, Ser A: 14, Pro A: 130

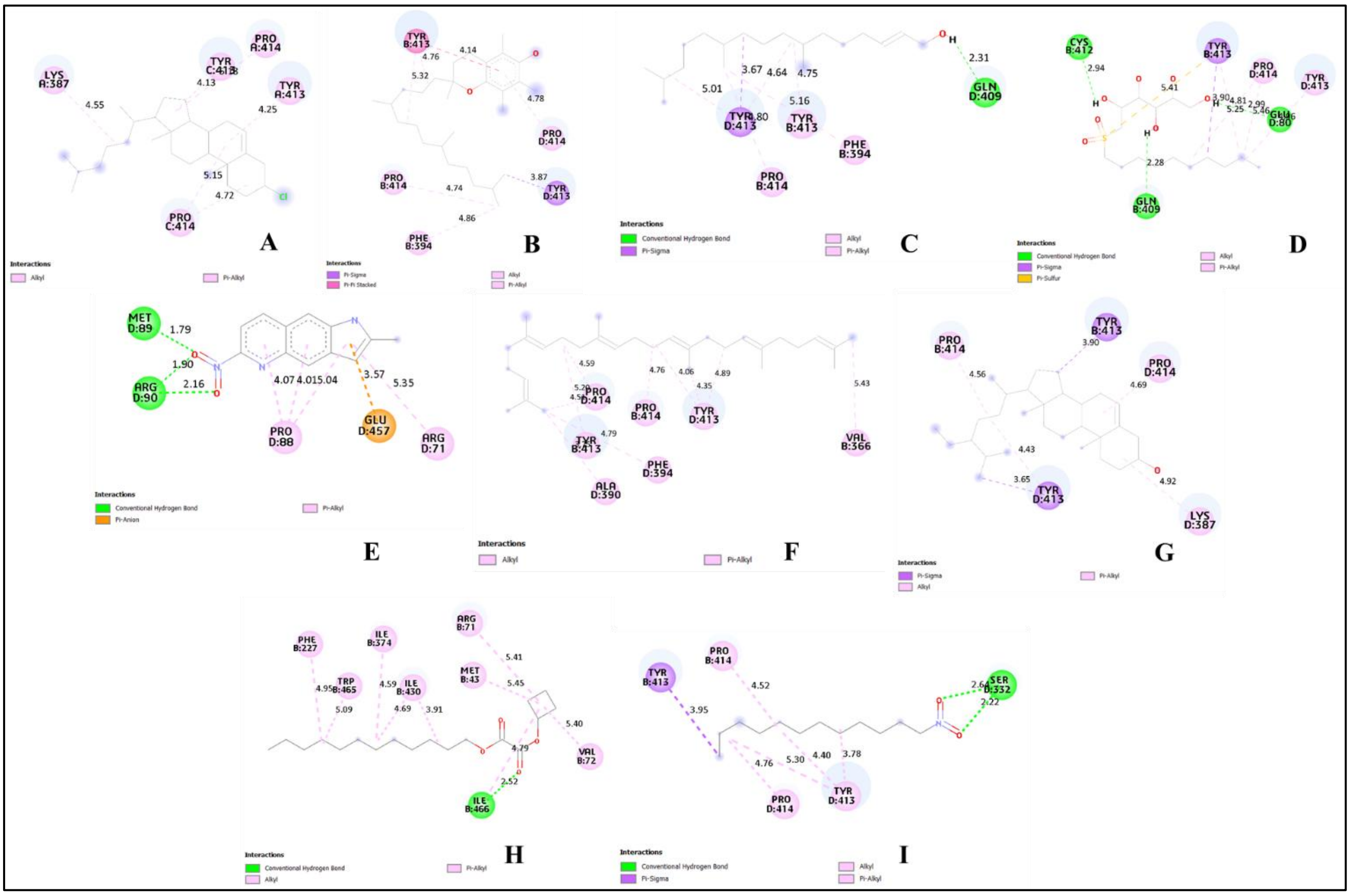

Figure 9 (A-I). 2D interaction diagrams of selected ligands from different extracts (A-3-chloro-5- cholestene, B- Vitamin E, C- Phytol, D- D-Mannitol, 1-Decylsulfonyl, E-6-Nitro-2-Methylpyrrolo[2,3-G] Quinoline, F- Squalene, G- Beta-Sitosterol, H- Oxalic acid, I- 1-Nitrododecane) with venom-related protein 3KVE showing hydrogen bonding, hydrophobic, and other key interactions.

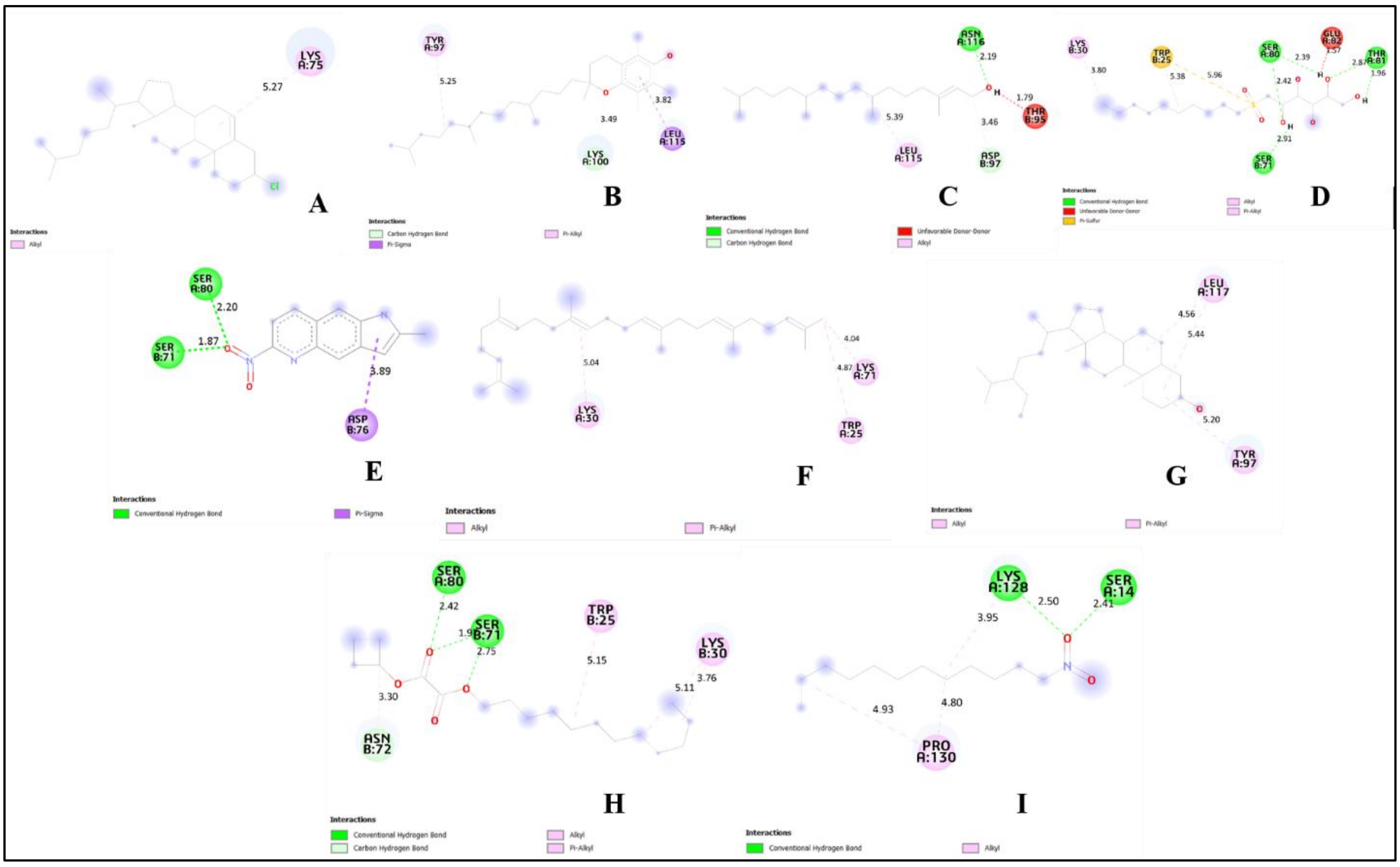

Figure 10 (A-I). 2D interaction diagrams of selected ligands from different extracts (A-3-chloro-5- cholestene, B- Vitamin E, C- Phytol, D- D-Mannitol, 1-Decylsulfonyl, E-6-Nitro-2-Methylpyrrolo[2,3-G] Quinoline, F- Squalene, G- Beta-Sitosterol, H- Oxalic acid, I- 1-Nitrododecane) with venom-related protein 3UBU showing hydrogen bonding, hydrophobic, and other key interactions.

In vitro validation

Table 5. Trypsin-casein Protease Inhibition assay

**Treatment**	**OD at 595 R1**	**OD at 595 R2**	**OD at 595 R3**	**OD at 595 R4**	**Mean**	**SD**	**Mean ± SD**	**% OF INHIBITION**
Positive control	0.234	0.236	0.237	0.242	0.23725	0.003403	0.2373 ± 0.0034	-
S1 (1mg/ml))	0.213	0.214	0.221	0.211	0.21475	0.004349	0.2148 ± 0.0043	19.60784314
S2 (2mg/ml))	0.203	0.204	0.209	0.202	0.2045	0.003109	0.2045 ± 0.0031	28.54030501
S3 (3mg/ml))	0.197	0.196	0.199	0.203	0.19875	0.003096	0.1988 ± 0.0031	33.55119826

S4 (4mg/ml))	0.183	0.189	0.184	0.181	0.18425	0.003403	0.1843 ± 0.0034	46.18736383
S5 (5mg/ml))	0.173	0.174	0.169	0.172	0.172	0.00216	0.1720 ± 0.0022	56.8627451
Negative control	0.125	0.119	0.124	0.122	0.1225	0.002646	0.1225 ± 0.0026	-

To calculate the Trypsin-casein Protease Inhibition percentage

$$\% \textbf{ Trypsin casein Protease Inhibition } = \frac{OD_{positive} - OD_{sample}}{OD_{positive} - OD_{negative}} \times 100$$

ANOVA results

Ordinary one-way ANOVA
ANOVA results

1	Table Analyzed	Data 1				
2	Data sets analyzed	A-F				
3						
4	**ANOVA summary**					
5	F	191.8				
6	P value	<0.0001				
7	P value summary	****				
8	Significant diff. among means (P < 0.05)?	Yes				
9	R square	0.9816				
10						
11	**Brown-Forsythe test**					
12	F (DFn, DFd)	0.1200 (5, 18)				
13	P value	0.9862				
14	P value summary	ns				
15	Are SDs significantly different (P < 0.05)?	No				
16						
17	**Bartlett's test**					
18	Bartlett's statistic (corrected)	1.272				
19	P value	0.9378				
20	P value summary	ns				
21	Are SDs significantly different (P < 0.05)?	No				
22						
23	**ANOVA table**	**SS**	**DF**	**MS**	**F (DFn, DFd)**	**P value**
24	Treatment (between columns)	0.01055	5	0.002110	F (5, 18) = 191.8	P<0.0001
25	Residual (within columns)	0.0001980	18	1.100e-005		
26	Total	0.01075	23			
27						
28	**Data summary**					
29	Number of treatments (columns)	6				
30	Number of values (total)	24				
31						

Ordinary one-way ANOVA output generated using GraphPad Prism for protease inhibition data

ANOVA results | Multiple comparisons

Ordinary one-way ANOVA
Multiple comparisons

Number of families	1
Number of comparisons per family	5
Alpha	0.05

Dunnett's multiple comparisons test	Mean Diff.	95.00% CI of diff.	Significant?	Summary	Adjusted P Value	A-?	
Positive control vs. S1 (1mg/ml))	0.02250	0.01602 to 0.02898	Yes	****	<0.0001	B	S1 (1mg/ml))
Positive control vs. S2 (2mg/ml))	0.03275	0.02627 to 0.03923	Yes	****	<0.0001	C	S2 (2mg/ml))
Positive control vs. S3 (3mg/ml))	0.03850	0.03202 to 0.04498	Yes	****	<0.0001	D	S3 (3mg/ml))
Positive control vs. S4 (4mg/ml))	0.05300	0.04652 to 0.05948	Yes	****	<0.0001	E	S4 (4mg/ml))
Positive control vs. S5 (5mg/ml))	0.06525	0.05877 to 0.07173	Yes	****	<0.0001	F	S5 (5mg/ml))

Test details	Mean 1	Mean 2	Mean Diff.	SE of diff.	n1	n2	q	DF
Positive control vs. S1 (1mg/ml))	0.2373	0.2148	0.02250	0.002345	4	4	9.594	18
Positive control vs. S2 (2mg/ml))	0.2373	0.2045	0.03275	0.002345	4	4	13.96	18
Positive control vs. S3 (3mg/ml))	0.2373	0.1988	0.03850	0.002345	4	4	16.42	18
Positive control vs. S4 (4mg/ml))	0.2373	0.1843	0.05300	0.002345	4	4	22.60	18
Positive control vs. S5 (5mg/ml))	0.2373	0.1720	0.06525	0.002345	4	4	27.82	18

Dunnett's multiple comparison analysis comparing extract-treated groups with the positive control of protease inhibition assay

Membrane stabilization assay

Table6. Raw absorbance values obtained from the erythrocyte membrane stabilization assay

SAMPLES	R1	R2	R3	MEAN	SD	% MEMBRANE STABILIZATION
Positive Control	0.252	0.245	0.249	0.248667	0.003512	
Negative Control	0.05	0.049	0.053	0.050667	0.002082	
S1 (1mg/ml)	0.213	0.21	0.209	0.210667	0.002082	19.19191919
S2 (2mg/ml)	0.199	0.202	0.201	0.200667	0.001528	24.24242424
S3 (3mg/ml)	0.183	0.19	0.188	0.187	0.003606	31.14478114
S4 (4mg/ml)	0.181	0.179	0.179	0.179667	0.001155	34.84848485
S5 (5mg/ml)	0.172	0.17	0.171	0.171	0.001	39.22558923

To calculate the % Membrane Stabilization

$$\%\text{Stabilization} = \frac{OD_{positive} - OD_{sample}}{OD_{positive} - OD_{negative}} \times 100$$

Ordinary one-way ANOVA<br>ANOVA results					
P value summary	****				
Significant diff. among means (P < 0.05)?	Yes				
R square	0.9942				
**Brown-Forsythe test**					
F (DFn, DFd)	0.6851 (5, 12)				
P value	0.6438				
P value summary	ns				
Are SDs significantly different (P < 0.05)?	No				
**Bartlett's test**					
Bartlett's statistic (corrected)					
P value					
P value summary					
Are SDs significantly different (P < 0.05)?					
**ANOVA table**	**SS**	**DF**	**MS**	**F (DFn, DFd)**	**P value**
Treatment (between columns)	0.01172	5	0.002343	F (5, 12) = 409.5	P<0.0001
Residual (within columns)	6.867e-005	12	5.722e-006		
Total	0.01178	17			
**Data summary**					
Number of treatments (columns)	6				
Number of values (total)	18				

Ordinary one-way ANOVA output generated using GraphPad Prism for membrane stabilization data

Ordinary one-way ANOVA<br>Multiple comparisons								
Number of families	1							
Number of comparisons per family	5							
Alpha	0.05							
**Dunnett's multiple comparisons test**	**Mean Diff.**	**95.00% CI of diff.**	**Significant?**	**Summary**	**Adjusted P Value**	**A-?**		
Positive control vs. S1(1mg/ml)	0.03800	0.03233 to 0.04367	Yes	****	<0.0001	B	S1(1mg/ml)	
Positive control vs. S2 (2mg/ml)	0.04800	0.04233 to 0.05367	Yes	****	<0.0001	C	S2 (2mg/ml)	
Positive control vs. S3 (3mg/ml))	0.06167	0.05600 to 0.06733	Yes	****	<0.0001	D	S3 (3mg/ml))	
Positive control vs. S4 (4mg/ml))	0.06900	0.06333 to 0.07467	Yes	****	<0.0001	E	S4 (4mg/ml))	
Positive control vs. S5 (5mg/ml))	0.07767	0.07200 to 0.08333	Yes	****	<0.0001	F	S5 (5mg/ml))	
**Test details**	**Mean 1**	**Mean 2**	**Mean Diff.**	**SE of diff.**	**n1**	**n2**	**q**	**DF**
Positive control vs. S1(1mg/ml)	0.2487	0.2107	0.03800	0.001953	3	3	19.46	12
Positive control vs. S2 (2mg/ml)	0.2487	0.2007	0.04800	0.001953	3	3	24.58	12
Positive control vs. S3 (3mg/ml))	0.2487	0.1870	0.06167	0.001953	3	3	31.57	12
Positive control vs. S4 (4mg/ml))	0.2487	0.1797	0.06900	0.001953	3	3	35.33	12
Positive control vs. S5 (5mg/ml))	0.2487	0.1710	0.07767	0.001953	3	3	39.76	12

Dunnett's multiple comparison analysis comparing extract-treated groups with the positive control.
